# Fatty acid oxidation drives acetyl-CoA-dependent H3K9ac reprogramming to promote adaptive resistance to BRAF^V600E^ inhibition in thyroid cancer

**DOI:** 10.1038/s41419-026-08575-7

**Published:** 2026-03-20

**Authors:** Xumeng Wang, Jing Zhang, Jimeng Yuan, Liping Wen, Tianxing Ying, Zehang Xu, Zheng Zhou, Shitu Chen, Quan Zhou, Jinghao Sheng, Chi Luo, Lisong Teng, Weibin Wang

**Affiliations:** 1 Department of Surgical Oncology of The First Affiliated Hospital, Zhejiang University School of Medicine, Hangzhou, China; 2 Department of Thoracic Surgery, Zhejiang Cancer Hospital, Hangzhou, China; 3 Hangzhou Institute of Medicine, Chinese Academy of Sciences, Hangzhou, China; 4 Institute of Immunology, Zhejiang University School of Medicine, Hangzhou, China; 5 Zhejiang Laboratory for Systems & Precision Medicine, Zhejiang University Medical Center, Hangzhou, China; 6 Institute of Environmental Medicine of Affiliated Hangzhou First People’s Hospital, Zhejiang University School of Medicine, Hangzhou, China; 7 Liangzhu Laboratory, Zhejiang University, Hangzhou, China

**Keywords:** Cancer metabolism, Targeted therapies

## Abstract

BRAF-targeted therapy is a promising strategy for thyroid cancer. However, its efficacy is limited by drug resistance. This study elucidates the role of fatty acid oxidation (FAO) in mediating adaptive resistance to BRAF^V600E^ inhibition (BRAFi) in thyroid cancer. Through integrated transcriptomic and metabolomic analyses, we demonstrate that BRAFi by vemurafenib (PLX4032) significantly enhances FAO in thyroid cancer cells. The pharmacological inhibition of FAO via thioridazine (Thio) synergizes with BRAFi to suppress tumor growth in vitro, in vivo and in a patient-derived organoid. Mechanistically, this metabolic shift is driven by the upregulation of PGC1α, which enhances FAO. The consequent increase in intracellular acetyl-CoA reprograms the histone H3K9 acetylation (H3K9ac) landscape, thereby epigenetically activating pro-survival genes such as RUNX1. In addition, higher expression of RUNX1 correlates with poorer prognosis in thyroid cancer. Consistently, functional studies confirm RUNX1’s oncogenic role, as its knockdown reduces cell proliferation, migration, and invasion. In conclusion, our work reveals a metabolic-epigenetic axis underlying adaptive response to BRAFi and identifies RUNX1 as a novel oncogene in thyroid cancer.

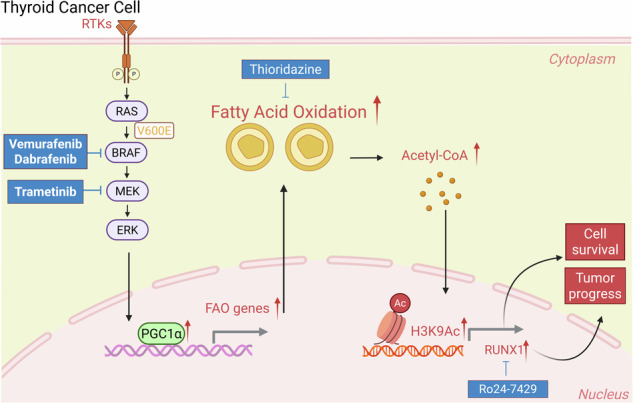

## Introduction

Thyroid cancer, recognized as the most prevalent endocrine malignancy, has seen a sustained increase in global incidence over recent decades [1]. Although the majority of cases exhibit indolent clinical behavior, aggressive histologic subtypes, particularly poorly differentiated (PDTC) and anaplastic thyroid cancer (ATC), marked by rapid locoregional invasion and dismal median overall survival (8–12 months), persist as critical therapeutic challenges [2, 3]. BRAF^V600E^ mutation is a driver oncogene for these patients, highlighting BRAF-targeted therapy as a cornerstone for managing advanced patients [4]. Nonetheless, BRAF-targeted agents achieve limited therapeutic efficacy due to the emergence of drug resistance. Several studies have attempted to clarify how BRAF-targeted resistance occurs, including BRAF splicing variants, activation of the RTK pathway [5–8]. We previously demonstrated that autophagy activation and VCAM-1 (vascular cell adhesion molecule 1) upregulation constitute key mediators of cellular adaptive responses to BRAF^V600E^ inhibition (BRAFi) [9, 10]. However, underlying mechanisms remain to be fully elucidated.

In recent years, altered lipid metabolism has garnered significant attention as a key contributor to tumor progression and drug resistance [11]. For instance, our analysis revealed that preoperative serum lipids can serve as novel predictors of thyroid cancer patients’ survival outcomes [12]. Fatty acid oxidation (FAO) represents another lipid metabolic pathway closely associated with drug resistance. This metabolic process involves the catabolism of long-chain fatty acids, enables cancer cells, particularly those under pharmacological treatment or within nutrient-deprived tumor microenvironments, to maintain bioenergetic and redox homeostasis, thereby promoting survival [13–15]. While FAO has attracted growing interest, its specific role in thyroid cancer remains poorly characterized. Furthermore, although existing studies have established correlations between FAO and drug responsiveness, the mechanisms by which FAO contributes to drug evasion are not fully understood.

In parallel, the interplay between tumor metabolism and epigenetic regulation has garnered increasing attention. Beyond its canonical roles in energy production and biomass generation, tumor metabolism exerts direct regulatory control over cellular signaling cascades and chromatin-mediated transcriptional programs [16]. Taking lactate, for instance, our prior work demonstrated that it could reconfigure the cellular lactylation landscape, thereby driving thyroid cancer proliferation via activating cell growth-related genes [17]. Acetyl-CoA, a key metabolic product of FAO, is a central node in cellular metabolism that bridges metabolism and epigenetics by serving as the essential substrate for histone acetylation, thereby activating oncogenic pathways and promoting tumor progression, such as proliferation and metastasis [18]. However, the metabolic-epigenetic crosstalk underlying BRAF-targeted therapy resistance remains to be fully elucidated.

Here, we systematically investigated metabolism reprogramming and relevant regulatory mechanisms during BRAFi in thyroid cancer. Notably, PGC1α-driven FAO functionally enhances global histone acetylation landscapes, particularly at H3K9 loci, which in turn epigenetically activate the expression of pro-survival genes, including RUNX1. Furthermore, we evaluated the therapeutic efficacy of combining BRAF and FAO inhibitors both in vitro and in vivo. Additionally, we investigated the potential role RUNX1 plays in thyroid cancer. We believe our research can offer novel avenues for BRAF-targeted therapy and provide new insights into thyroid cancer progression.

## Methods

### Patients and clinical specimens

Thyroid cancer and para-cancerous tissue samples were randomly collected from 88 patients who underwent their first thyroidectomy between 2020 and 2022 in the First Affiliated Hospital of Zhejiang University (Hangzhou, China). All tissue samples were retrieved from formalin-fixed paraffin-embedded blocks and reviewed by an experienced attending pathologist. All patients were informed that their specimens were stored by the hospital and potentially used for scientific research, and research-informed consents were obtained. The study was approved by the Medical Ethics Committee of the First Affiliated Hospital of Zhejiang University (reference number: 2025B0851).

### Cell culture

Anaplastic thyroid cancer cell lines: 8505c was purchased from DSMZ (Braunschweig, Germany). WRO was kindly provided by Dr James A. Fagin (Memorial Sloan-Kettering Cancer Institute, New York, USA). BHT-101 and CAL-62 were purchased from the Cell Bank of the Chinese Academy of Sciences (Shanghai, China). Papillary thyroid cancer cell line BCPAP was purchased from DSMZ. KTC1 was purchased from the Cell Bank of the Chinese Academy of Sciences. The normal thyroid gland cell Nthy-ori-3-1 was kindly provided by Pro. Tiannan Guo (Westlake University, Zhejiang, China). 8505c, WRO, BHT-101 and BCPAP harbor BRAF^V600E^ mutatiion. The cells were cultured in RPMI1640 medium (Gibco, Rockville, USA), except for BHT-01, which was cultured in DMEM medium (Gibco, Rockville, USA). The PLX4032-resistant 8505c cells were cultured in RPMI1640 medium containing 2.5 μM PLX4032. All the cells were supplemented with 10% fetal bovine serum (TIANHANG, Zhejiang, China) and antibiotics (100 U/ml penicillin and 100 mg/ml streptomycin) at 37 °C in a humidified incubator (PHCbi, Osaka, Japan) with 5% CO_2_. All the cell lines have undergone Sanger sequencing for identifying BRAF^V600E^ mutation, and corresponding STR have been performed for cell line authentication.

### Chromatin immunoprecipitation (ChIP) and following sequence (ChIP-seq)

Cells at 70–80% confluence were fixed with the help of 1% formaldehyde to preserve DNA-protein interactions. Then the cells were mixed with glycine until the final concentration reached 0.15 M to stop the cross-linking. Subsequent to crosslinking, the cells were randomly broken by an 18-cycle ultrasonic wave treatment (20% power, 5’s on, 5’s off each time), which was then followed by centrifugation at 4 °C and 10 000 g for 10 min. The resultant supernatants were collected and incubated with 2 μg of Acetyl-Histone H3 (Lys9) antibodies (PTM BIO) to bind DNA regions at 4 °C overnight. The next day, protein-DNA was pulled down by 40 μl protein A agarose beads (Invitrogen, California, USA) at 4 °C for 2 h, followed by sequential washes with low-salt (0.1% SDS, 1% Triton X-100), high-salt (500 mM NaCl), and LiCl buffers. After de-crosslinking, the DNA was purified using the phenol-chloroform method and ethanol precipitation. Enrichment of target loci was verified by qPCR using SYBR Green Supermix (Roche, Basel, Switzerland) to confirm the success of the ChIP assay. Purified DNA fragments were constructed, added to ChIP-seq libraries, amplified, and sequenced using the Illumina platform. The primers used for ChIP-qPCR are listed in the Supplementary material Table S3.

### Metabonomic profiling

Cells were first treated with different drugs for 24 h, then cells were placed on ice, and gently washed with PBS three times. Then, cells were collected, and after vortexing and centrifugation, supernatants were dried by centrifugal concentrator (Genevac miVac, UK). Following, metabolites were extracted using 1% acetonitrile, and supernatants were analyzed via ultra-high-performance liquid chromatography (UHPLC; Agilent 1290 II, Agilent Technologies, Germany) coupled with a high-resolution mass spectrometer (HR-MS; 5600 Triple TOF Plus, AB Sciex, Singapore). MS data were acquired in both positive and negative ionization modes. Raw data were processed using MarkerView 1.3 (AB Sciex, Concord, ON, Canada) software to extract peak areas, mass-to-charge ratios, and retention times, generating a two-dimensional data matrix. PeakView 2.2 (AB Sciex, Concord, ON, Canada) was employed to match secondary mass spectrometry data against Metabolites databases, HMDB, METLIN, and standards for metabolite identification. The identified metabolite IDs were then mapped back to corresponding ions in the primary mass spectrometry two-dimensional data matrix. R language-based programs were utilized for pathway analysis of the identified metabolomics data.

### ^13^C oleic acid labeling and metabolic flux analysis

For stable isotope tracing, the ^13^C oleic acid was first conjugated with 4 mM bovine serum albumin (BSA). Cells were cultured in 10 cm dishes. When they reached 70% confluency, the medium was replaced with fresh medium containing 100 μM conjugated ^13^C oleic acid with DMSO or 5 μM PLX4032 for 24 h. Then the cells were placed on ice, and gently washed with ice-cold PBS for three times. Then, cells were collected, resuspended in 200 μL of 80% methanol and sonicated for metabolite extraction. Then 800 μL of 80% methanol was added, after vortexing for 30 s and centrifuging at 14,000 × *g* (4 °C, 10 min), supernatant was transferred to a fresh 2 mL microcentrifuge tube, dried by centrifugal concentrator (Genevac miVac, UK), and redissolved in 100 μL 20% H₂O/80% methanol. After vortexing for 30 s followed by sonication, the supernatant was collected for further LC-MS/MS analysis (Thermo Vanquish Flex/QE-HF-X, USA). Raw Data processing was performed using Skyline (v21.1) and MAVEN (v3.0) for peak extraction, integration, and isotopic peak correction.

### RNA sequence analysis

Total RNA was extracted with TRIZOL reagent, and the total amounts and integrity of RNA were assessed using the RNA Nano 6000 Assay Kit of the Bioanalyzer 2100 system (Agilent Technologies, California, USA). cDNA libraries were generated and sequenced on an Illumina NovaSeq 6000 platform. The image data measured by the high-throughput sequencer is converted into sequence data (reads) by CASAVA base recognition. The index of the reference genome was built using Hisat2 (v2.0.5), and paired-end clean reads were aligned. FeatureCounts (v1.5.0-p3) was used to count the number of reads mapped to each gene. Then, FPKM of each gene was calculated based on the length of the gene and the read count mapped to this gene. Finally, differential expression analysis was performed using the DESeq2 R package (1.20.0).

### Xenograft experiments

All in vivo studies were approved by the Animal Experimental Ethical Inspection of the First Affiliated Hospital, Zhejiang University School of Medicine (reference number: 2019332). Six-week-old female nude mice were used in this study, and tumor xenografts were established by injection of 5 × 10^6^ 8505c cells with 3:1 Matrigel (Corning, New York, USA) into the flank region of the mice. Ten days after implantation, when the tumor became palpable, mice were randomly assigned to different groups, and the following treatments were carried out: negative control (vehicle group), 20 mg/kg Thio group, 30 mg/kg PLX4032 group, and combined group. The drugs were first dissolved in DMSO, and suspended to the indicated concentration (formulated with 10% tween-20, 40% Polyethylene glycol, 50% sterile water). PLX4032 was administered orally once daily, and Thio was administered by intraperitoneal injection every two days for 16 days. Tumor size measurements were performed every other day. Tumor volume was calculated using the formula *V* = AB^2^/2, where A and B are the long and short diameters of the tumor, respectively; tumors were harvested at the end of the in vivo experiments and tumor weights were measured.

### Organoid establishment and drug response test

Fresh tumor specimens were obtained from a papillary thyroid cancer patient treated at the First Affiliated Hospital of Zhejiang University following written informed consent. All procedures received institutional review board approval from the Clinical Research Ethics Committee of the First Affiliated Hospital of Zhejiang University School of Medicine (reference number: IIT20250806A). Gene detection was performed to confirm BRAF^V600E^ mutation. To generate organoids, shredded patient-derived tumor tissues were dissociated into single cells through mechanical dissociation and enzymatic digestion. The cells were then suspended in Matrigel-enriched organoid growth medium, and concentrations were quantified using dual-chamber hemocytometers. Next, the cells were seeded into 96-well ultra-low attachment plates at 7 × 10^3^ cells/well, and cultured in the advanced medium supplemented with growth factors and organoid-Matrigel under 5% CO₂ at 37 °C. At day 10 post-seeding, for drug sensitivity assays, organoids were exposed to different agents for drug sensitivity assay. Following 7-day exposure, viability was quantified via the Countstar Castor S1 3D Analyzer (Alit Biotech, Shanghai, China) using ATP-based bioluminescence normalized to vehicle controls.

### Statistical analysis

All experiments were repeated at least three times. Quantitative data are presented as mean ± SD. Differences between groups were assessed with the Student’s *t* test or ANOVA analysis and were considered statistically significant at *P* < 0.05 and highly significant at *P* < 0.01. Data were analyzed using GraphPad Prism 8.0 and SPSS 29.0.

### Additional methods and reagents

Details of the other methods and reagents used in this study are provided in the Supplementary material.

## Results

### Inhibition of BRAF^V600E^ induces fatty acid oxidation in thyroid cancer cells

**Fig. 1:**
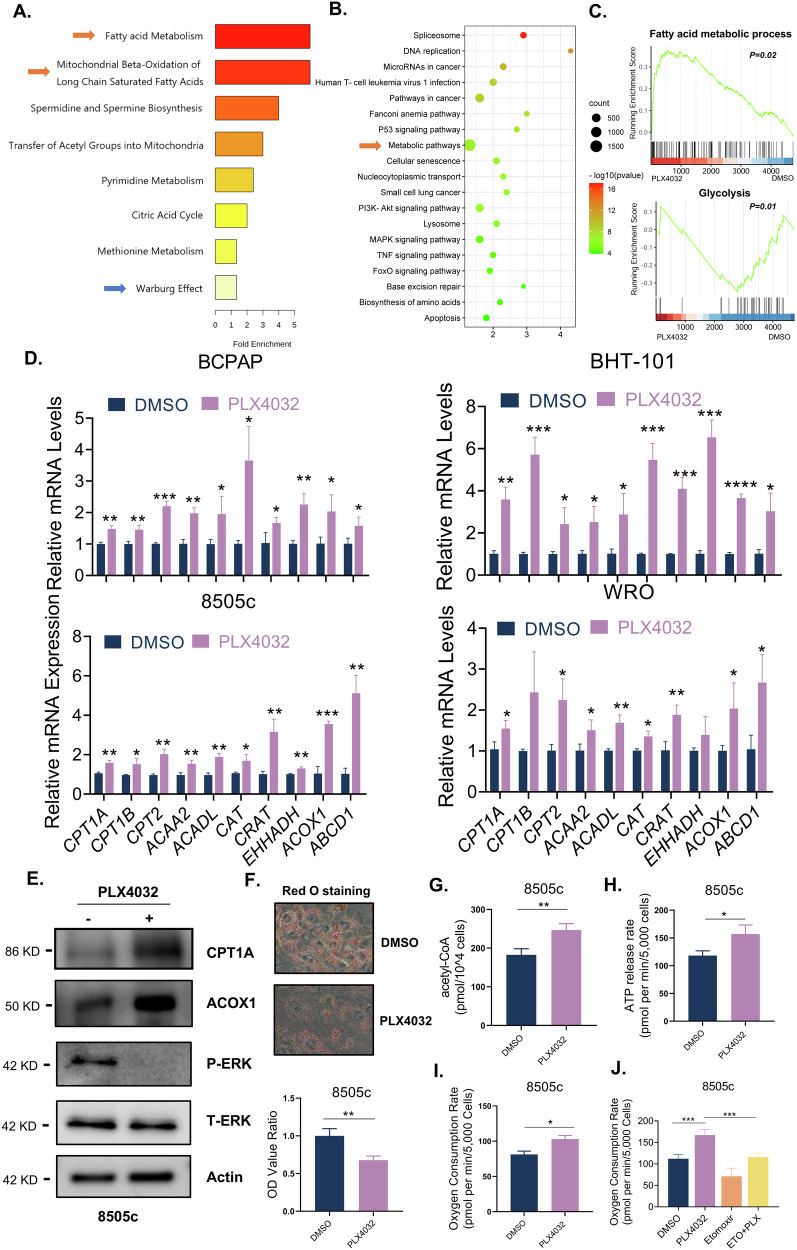
Inhibition of BRAF^V600E^ induces fatty acid oxidation in thyroid cancer cells. **A** Metabolite enrichment analysis of differentially metabolites after PLX4032 (5 μM, 24 h) treatment. **B** KEGG enrichment analysis of differentially expressed genes identified by RNA-seq after PLX4032 (5 μM, 24 h) treatment. **C** GSEA plots evaluating the changes in glycolysis and fatty acid metabolism. **D** BRAF-positive thyroid cancer cells were treated with DMSO or PLX4032 (5 μM) for 24 h, and followed by RT-qPCR detection of FAO-related genes. **E** Western blot analysis of CPT1A and ACOX1 in 8505c after PLX4032 (5 μM, 24 h) treatment. **F** Cellular lipid droplet was quantified via Oil Red O staining and followed by spectrophotometric measurement of optical density (OD) in 8505c after PLX4032(5 μM, 24 h) treatment. Cellular acetyl-CoA content (**G**) and ATP release rate (**H**) were determined by assay kits, oxygen consumption rate (OCR) was measured by oxygen respirometry system (**I**) in 8505c after PLX4032(5 μM, 24 h) treatment. **J** 8505c was treated with 5 μM PLX4032, 50 μM etomoxir respectively or the combination for 24 h, and followed by oxygen consumption detection. Data are presented as means ± s.d. Statistical significance was determined by Student’s *t* test in (**D**) and (**F**–**I**) analysis of variance (ANOVA) in J, with *P* ≤ 0.05 considered significant. *P < 0.05, **P < 0.01, ***P < 0.005 and ****P < 0.001.

BRAF-targeted therapy has emerged as a pivotal therapeutic approach for thyroid cancer, while drug resistance has limited its application. To elucidate the molecular mechanisms underlying the therapeutic response, we conducted sequential multi-omics profiling in 8505c cells following BRAF^V600E^ inhibition with vemurafenib (PLX4032). Metabolomic analysis revealed several metabolic pathway alterations, including glycolysis and fatty acid metabolism. Notably, the mitochondrial beta-oxidation of long-chain saturated fatty acids demonstrated significant alteration (Fig. 1A). Further, we performed RNA sequencing analysis. The volcano plot analysis identified total of 4203 significantly changed genes (Fig. S1A), Kyoto Encyclopedia of Genes and Genomes (KEGG) analysis also revealed metabolic processes as one of the most significantly altered pathways during BRAF inhibition, demonstrating the highest number of differentially expressed genes (Fig. 1B). Subsequent Gene Set Enrichment Analysis (GSEA) also identified the alterations of FAO and glycolysis (Fig. 1C), with key regulatory genes detailed listed in Supplementary Fig. 1B. Contrary to our initial observation of glycolysis inhibition, integrated multi-omics analysis revealed a compensatory activation of FAO. This metabolic plasticity indicates that tumors adapt to BRAF-targeted therapy in thyroid cancer by switching their energy substrate utilization.

To further validate these findings, we selected a panel of FAO-related genes for RT-qPCR validation across four BRAF^V600E^ mutant thyroid cancer cell lines (BCPAP, 8505c, WRO, and BHT-101) following PLX4032 treatment. The results confirmed that these genes were uniformly upregulated across all tested cell lines (Fig. 1D). Given that CPT1A and ACOX1 are two rate-limiting enzymes of FAO, we selected them for further validation and confirmed the protein level upregulation of both enzymes (Fig. 1E). Since lipid droplets provide essential substrates for FAO, we quantified their content using Oil Red O staining, and the analysis revealed that PLX4032 treatment induced a significant reduction in lipid droplet accumulation (Fig. 1F), suggesting rapid utilization of lipid for FAO during BRAFi. Meanwhile, acetyl-CoA, the direct product of FAO, showed increased levels after BRAFi (Fig. 1G). As FAO is an oxygen-consuming and ATP-generating process, we also measured these two functional indicators. Our results showed that PLX4032 significantly enhanced both oxygen consumption rate (OCR) and ATP production (Fig. 1H, I), whereas co-treatment with the FAO inhibitor etomoxir attenuated these effects (Fig. 1J). Oxidative phosphorylation (OXPHOS) represents another potential OCR source; we also detected OXPHOS-related gene expression after BRAFi, but these genes were not uniformly upregulated, with most remaining unchanged (Fig. S1C, D), indicating that FAO serves as the predominant contributor to OCR in our model. Considering mitochondria being a primary site of FAO, we evaluated mitochondrial activity by visualizing mitochondrial networks using Mito-Tracker staining. PLX4032 increased Mito-Tracker fluorescence intensity, indicating enhanced mitochondrial activity (Fig. S1E, F). Collectively, these results demonstrate that BRAFi activates FAO in thyroid cancer.

To further confirm whether FAO up-regulation represents a transient adaptation or sustained resistance mechanism, we established PLX4032-resistant 8505c cells (Fig. S2A). Consistently, expression profiling revealed persistent upregulation of FAO-related genes in resistant cells (Fig. S2B), suggesting that FAO activation serves as both an acute stress response and a durable survival mechanism during prolonged BRAFi exposure.

### Targeting FAO enhances the antitumor effect of BRAFi in thyroid cancer across preclinical models

**Fig. 2:**
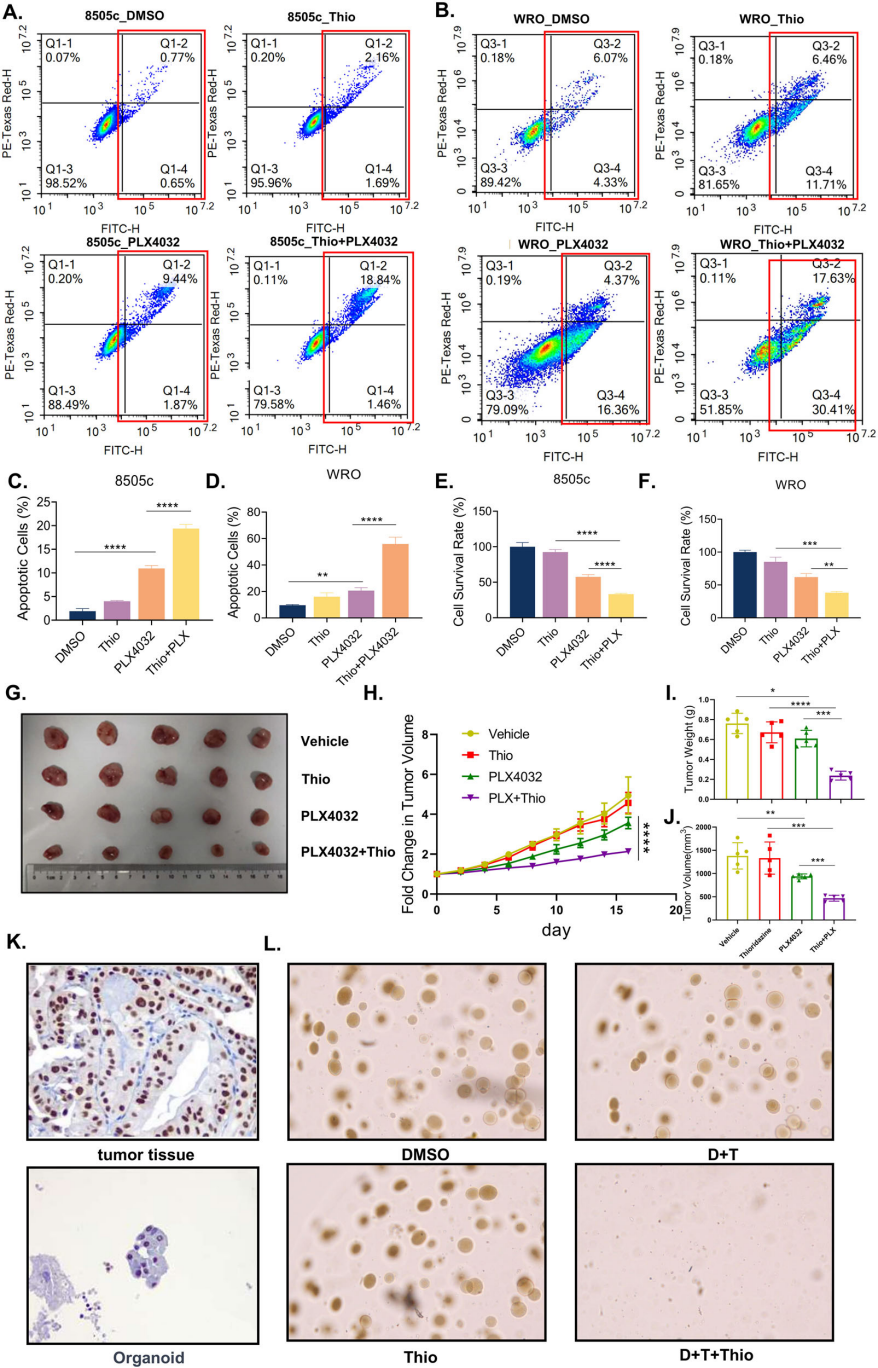
Targeting FAO enhances the antitumor effect of BRAFi in thyroid cancer across preclinical models. 8505c and WRO cells were treated with 5 μM BRAF^V600E^ inhibitor PLX4032 alone, 10 μM FAO inhibitor Thio alone or a combination of PLX4032 with Thio for 48 h, followed by cell apoptosis assay via the flow cytometry (**A**–**D**) cell number counting via cell counter (**E**, **F**) and corresponding statistical analysis. **G**–**I** Nude mice bearing 8505c xenografts were treated with vehicle, Thio (20 mg/kg per day), PLX4032 (30 mg/kg per day), or a combination of Thio and PL4032 for total 16 days. After drug administration at the last day, mice were sacrificed, and tumor volume and weight were measured (*n* = 5 for each group). **K** Hematoxylin-Eosin (HE) staining of thyroid-specific transcription factor TTF-1 in primary thyroid cancer tissue and constructed organoid (40X). **L** Thyroid cancer PDO was treated with dual 250 nM BRAFV600E inhibitor dabrafenib plus 10 nM MEK inhibitor trametinib (D + T), 10 μM Thio alone or triple combination therapy (D + T+Thio) for 7 days, followed by Countstar Castor S1 picture capture (20X). Data are presented as means ± s.d. Statistical significance was determined by ANOVA in (**C**–**F**) and (**H**–**J**), with *P* ≤ 0.05 considered significant. *P < 0.05, **P < 0.01, ***P < 0.005 and ****P < 0.001.

To investigate whether suppression of FAO potentiates BRAFi efficacy, we treated two BRAF^V600E^ mutant ATC cells, 8505c and WRO, with PLX4032 in combination with thioridazine (Thio), a pharmacological agent known to inhibit ACOX1, the rate-limiting enzyme of FAO. While PLX4032 monotherapy induced moderate apoptosis and Thio alone exhibited minimal cytotoxicity, their combined administration synergistically increased apoptotic cell death (Fig. 2A–D). Consistently, cell viability assays demonstrated a significantly greater reduction in surviving cell counts with combination therapy compared to single-agent treatments (Fig. 2E, F). In another PTC cell BCPAP, the combination of Thio with PLX4032 also achieved a remarkably cytotoxic effect (Fig. S4A). To validate these findings in vivo, tumor xenografts were established in BALB/c nude mice through subcutaneous injection of 8505c cells. Following systemic administration of therapeutic agents, the combinatorial regimen of PLX4032 and Thio exhibited superior antitumor efficacy, as evidenced by the reduction in both tumor volume and weight, whereas PLX4032 monotherapy achieved only moderate tumor burden reduction (Fig. 2G–J).

To further strengthen our conclusions, we treated 8505c cells with another BRAF inhibitor (PLX4720). Similarly, we observed upregulation of FAO-related genes (Fig. S2C) after PLX4720 treatment, and the cytotoxicity test also proved that Thio combined with PLX4720 remarkably reduced survival cell numbers compared to PLX4720 monotherapy (Fig. S4B). Notably, there are clinical trials evaluating BRAF inhibitor (Dabrafenib, D) plus MEK inhibitor (Trametinib, T) in advanced BRAF^V600E^ mutant thyroid cancer, which also demonstrated limited therapeutic efficacy [19]. We validated our findings in D + T-treated 8505c cells. Consistent with PLX4032 and PLX4720, concurrent D + T treatment induced substantial upregulation of FAO-related genes (Fig. S2D), which aligns with prior observation in melanoma [20], indicating FAO activation represents a conserved adaptive mechanism across MAPK-targeted therapies. To further assess clinical translatability, we established a patient-derived thyroid cancer organoid (PDO) model. Following histopathological validation of tumor characteristics recapitulation (Fig. 2K), drug sensitivity assays revealed that triple combination therapy (Thio+D + T) achieved significantly greater tumor growth inhibition relative to the D + T dual therapy (Fig. 2L). These results collectively demonstrate that pharmacological inhibition of FAO via Thio enhances MAPK-targeted therapy efficacy across preclinical models of thyroid cancer.

### Activation of PGC1α drives FAO during BRAFi in thyroid cancer cells

**Fig. 3:**
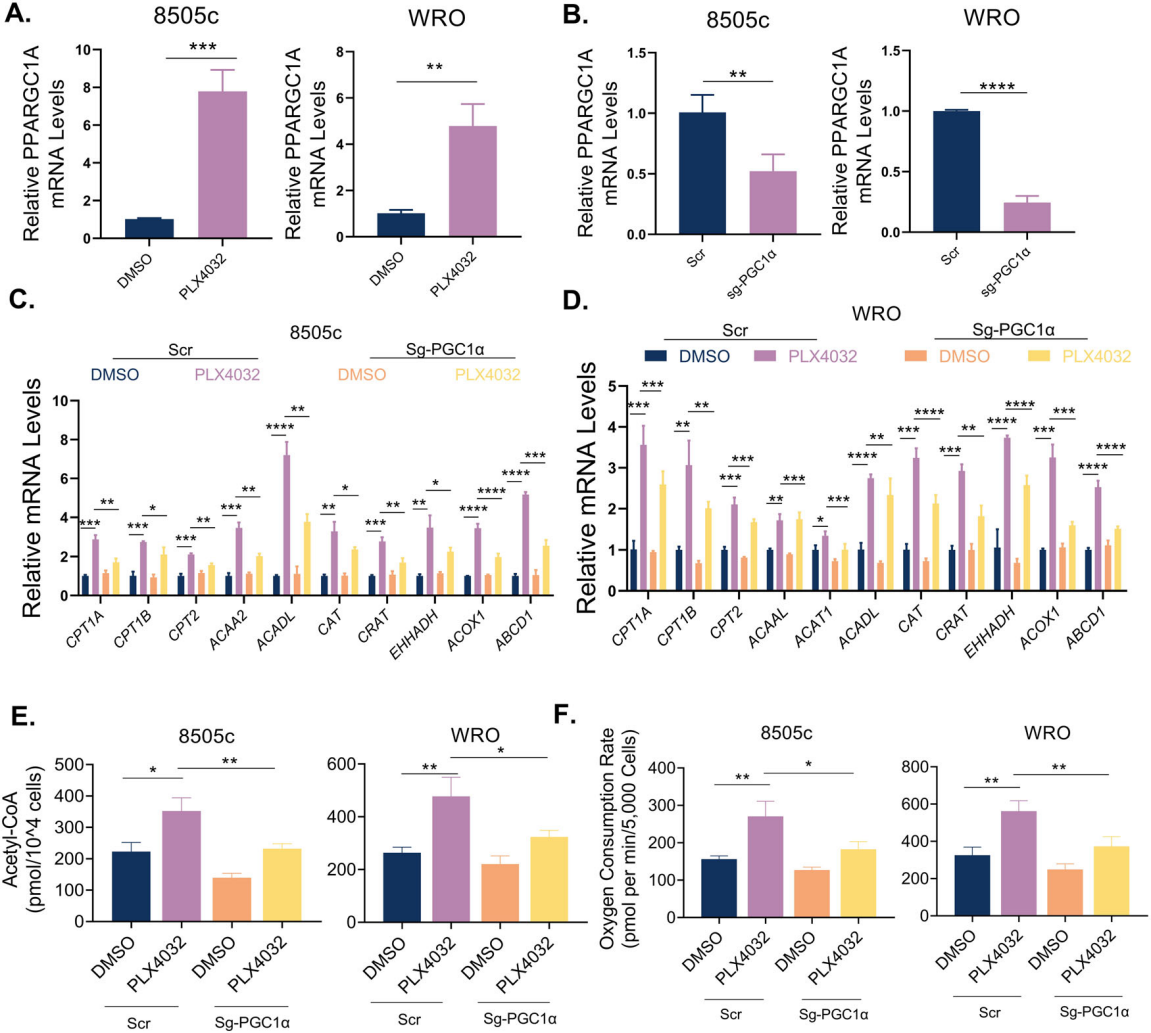
Activation of PGC1α drives FAO during BRAFi in thyroid cancer cells. **A** 8505c and WRO cells were treated with PLX4032 (5 μM, 24 h) and followed by RT-qPCR detection of PGC1α. **B** Knock-down efficiency invalidation of PGC1α in 8505c and WRO cells. **C**, **D** Vehicle and PGC1α-KD cells were treated with DMSO or PLX4032 (5 μM) for 24 h, then fatty acid oxidation related genes were detected by RT-qPCR. Vehicle and PGC1α-KD cells were treated with DMSO or PLX4032 (5 μM) for 24 h, then acetyl-CoA content was measured by acetyl-CoA fluorometric assay kit (**E**) and OCR was measured by oxygen respirometry system (**F**). Data are presented as means ± s.d. Statistical significance was determined by Student’s *t* test in (**A**) and (**B**) ANOVA in (**C**–**F**) with *P* ≤ 0.05 considered significant. *P < 0.05, **P < 0.01, ***P < 0.005 and ****P < 0.001.

PPARGC1A (peroxisome proliferator-activated receptor gamma coactivator 1 alpha, PGC1α) is recognized as a central transcriptional coactivator governing cellular energy metabolism [21]. To elucidate whether PGC1α orchestrates FAO activation during BRAFi, we first examined PGC1α expression and found that it was significantly upregulated following PLX4032 treatment in both 8505c and WRO cells (Fig. 3A). To investigate its functional relevance, we generated stable PGC1α knockdown (PGC1α-KD) cells. Following confirmation of knockdown efficiency (Fig. 3B), both control and PGC1α-KD cells were treated with PLX4032. Notably, PGC1α depletion significantly attenuated BRAFi-induced transcriptional activation of FAO-related genes (Fig. 3C, D), abrogated acetyl-CoA accumulation (Fig. 3E), and blunted OCR elevation (Fig. 3F). Pharmacological inhibition of PGC1α using SR-18292 (a selective PGC1α antagonist) prior to BRAFi also recapitulated the genetic perturbation, suppressing FAO gene expression (Fig. S3A, B), limiting acetyl-CoA production (Fig. S3C), and attenuating OCR enhancement (Fig. S3D).

**Fig. 4:**
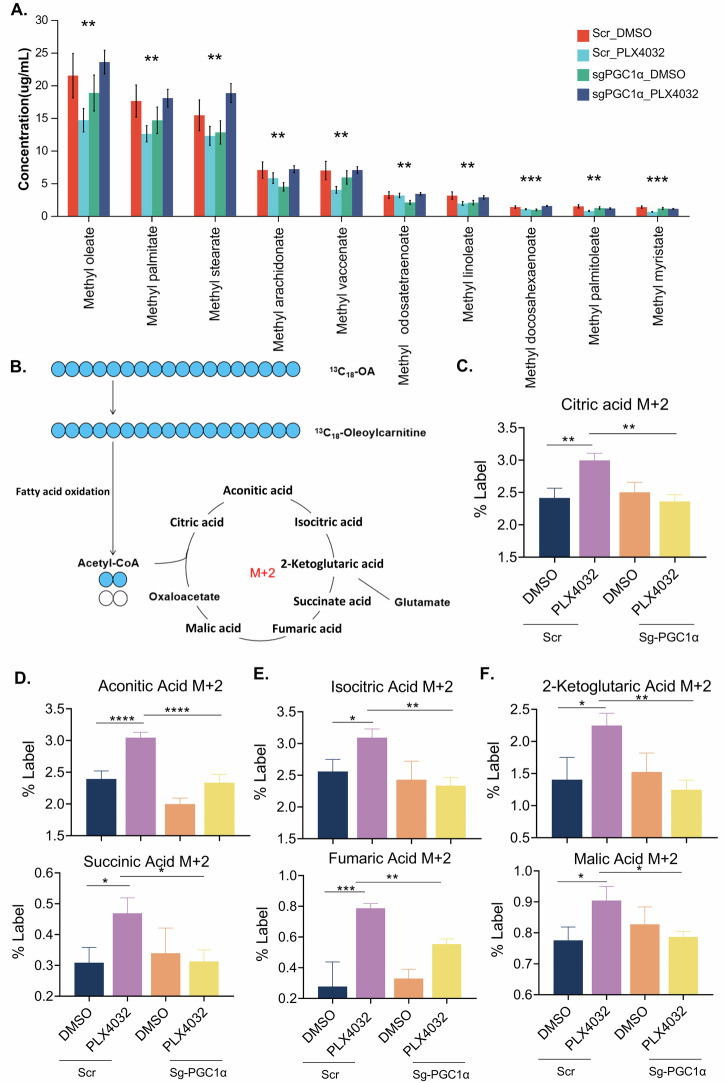
Metabolic flux reveals PGC1α knockdown attenuated BRAFi-induced FAO enhancement in thyroid cancer. **A** Vehicle and PGC1α-KD cells were treated with DMSO or 5 μM PLX4032 for 24 h, then cells were collected for targeted metabonomic analysis. **B** Vehicle and PGC1α-KD cells were treated with 100 μM conjugated ^13^C oleic acid DMSO or PLX4032 (5 μM) for 24 h, then metabolites were collected for LC/MS analysis. Each cycle of FAO generates one acetyl-CoA molecule containing two labeled carbon atoms (M + 2), which subsequently enters the tricarboxylic acid (TCA) cycle. **C**–**F** Metabolite mass isotopic integration in m + 2 of each metabolite is presented by comparing the fractions of relative abundance of ^13^C isotopes. Data are presented as means ± s.d. Statistical significance was determined by ANOVA in A, C-F, with *P* ≤ 0.05 considered significant. *P < 0.05, **P < 0.01, ***P < 0.005 and ****P < 0.001.

FAO is a metabolic process that utilizes medium- and long-chain fatty acids as substrates to generate acetyl-CoA and cellular energy. To precisely assess FAO activity, we performed targeted metabolomic analysis of these fatty acids in 8505c cells. The results revealed that PLX4032 treatment induced a significant reduction in medium- and long-chain fatty acid levels, suggesting rapid transport of these fatty acids for FAO utilization. In contrast, PGC1α knockdown groups exhibited accumulation of these fatty acids despite PLX4032 treatment, suggesting impaired fatty acid consumption and FAO capacity (Fig. 4A). Moreover, to further directly evaluate FAO activity, we performed isotope tracer metabolic flux using ^13^C-labeled oleic acid as the substrate in 8505c cells. Each cycle of FAO generates one acetyl-CoA molecule containing two labeled carbon atoms (M + 2), which subsequently enters the tricarboxylic acid (TCA) cycle. Therefore, the relative abundance of labeled TCA cycle intermediates (M + 2) serves as a precise indicator of FAO activity (Fig. 4B). Consistently, the results found that PLX4032 significantly increased FAO-related intermediates in control groups, whereas PGC1α knockdown abolished this effect (Fig. 4C–F). Collectively, both genetic and pharmacological interventions substantiated that PGC1α activation is mechanistically required for the BRAFi-induced FAO upregulation in thyroid cancer.

### Altered FAO metabolites following BRAFi reprogram H3K9 acetylation landscape in thyroid cancer cells

**Fig. 5:**
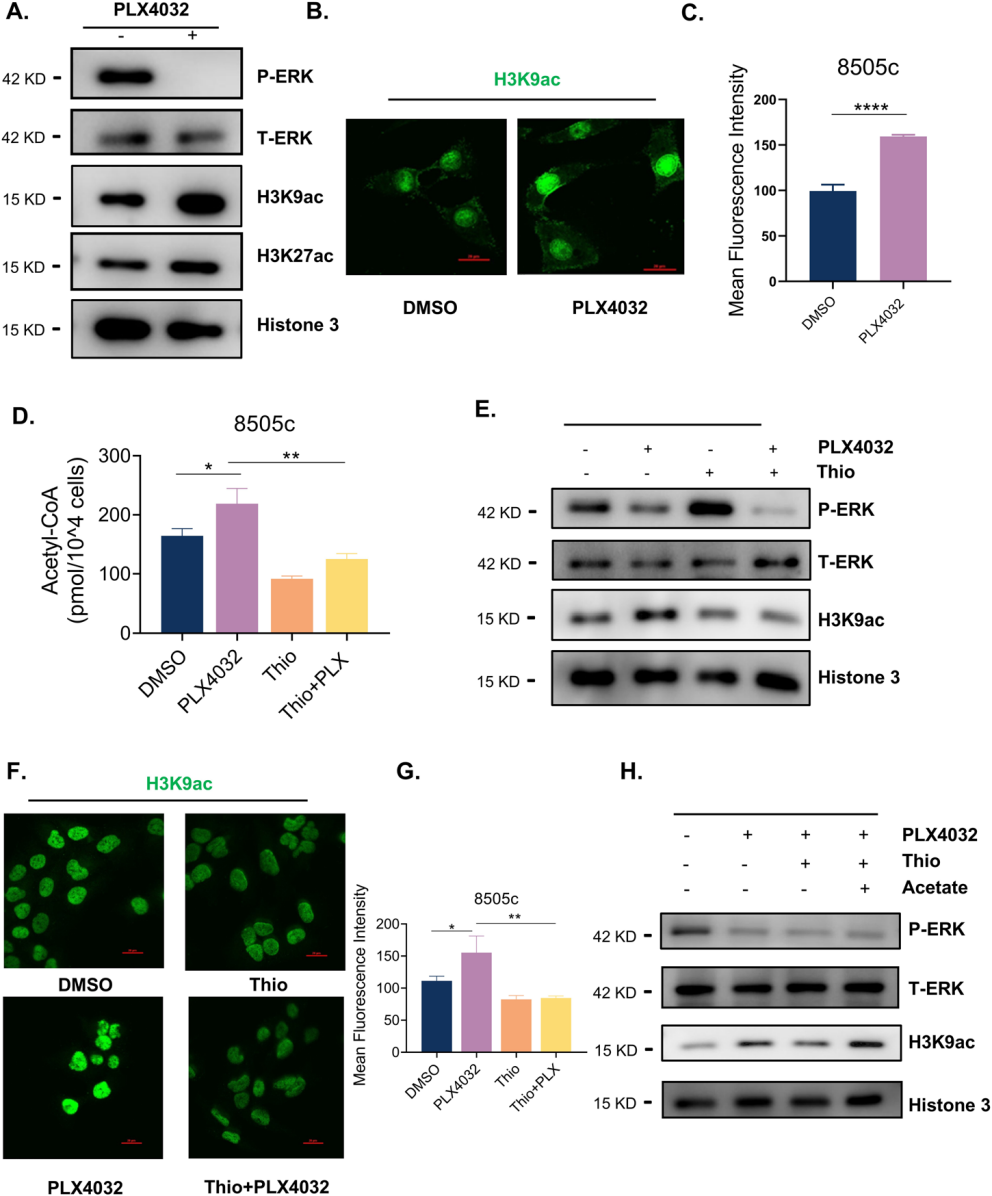
Altered FAO metabolites following BRAFi reprogram H3K9 acetylation landscape in thyroid cancer cells. **A** Histone acetylation including H3K9ac and H3K27ac were detected after PLX4032 (5 μM, 24 h) treatment by western blot in 8505c. **B**, **C** H3K9ac were detected after PLX4032 (5 μM, 24 h) treatment by immunofluorescence in 8505c, and corresponding statistical analysis of the immunofluorescence intensity. **D** 8505c cells were treated with PLX4032 5 μM / Thio 10 μM independently or together for 24 h, then acetyl-CoA content was measured by acetyl-CoA fluorometric assay kit. **E**, **F** Western blot and immunofluorescence detection of H3K9ac in the presence or absence of 10 μM Thio alongside PLX4032 (5 μM) for 24 h in 8505c. **G** Statistical analysis of the immunofluorescence intensity. **H** H3K9ac was detected by western blot after acetate supplementation (10 mM) in combination with of PLX4032 (10 μM) and Thio (10 μM) treatment for 24 h in 8505c cells. Data are presented as means ± s.d. Statistical significance was determined by Student’s *t* test in C, ANOVA in D and G, with *P* ≤ 0.05 considered significant. *P < 0.05, **P < 0.01, ***P < 0.005 and ****P < 0.001.

To further investigate how FAO promotes thyroid cancer cell survival during BRAFi, we focused on acetyl-CoA, the intermediate metabolic product of FAO, which is an essential substrate for histone acetylation. Building on our earlier findings that elevated intracellular acetyl-CoA levels induced by PLX4032 in Fig. 2, we assessed the dynamics of histone acetylation. Western blot analysis demonstrated PLX4032 enhanced acetylation (ac) at both H3K9 and H3K27 sites, with H3K9ac exhibiting more pronounced enrichment (Fig. 5A). This observation was further confirmed by immunofluorescence (Fig. 5B, C). To establish the functional link between FAO-derived acetyl-CoA and epigenetic remodeling, we co-treated 8505c with Thio and PLX4032. The combination treatment partially reversed the PLX4032 induced accumulation of acetyl-CoA (Fig. 5D) and led to a concomitant attenuation of H3K9ac level (Fig. 5E–G). We then performed rescue experiments by supplementing exogenous sodium acetate (a direct acetyl-CoA precursor) to cells treated with PLX4032 and Thio, which partially restored H3K9ac level (Fig. 5H). Together, these data demonstrate that BRAFi drives FAO-dependent acetyl-CoA accumulation, thereby promoting histone hyperacetylation, particularly H3K9ac.

### Enhanced H3K9ac facilitates pro-survival gene expression upon BRAFi in thyroid cancer cells

**Fig. 6:**
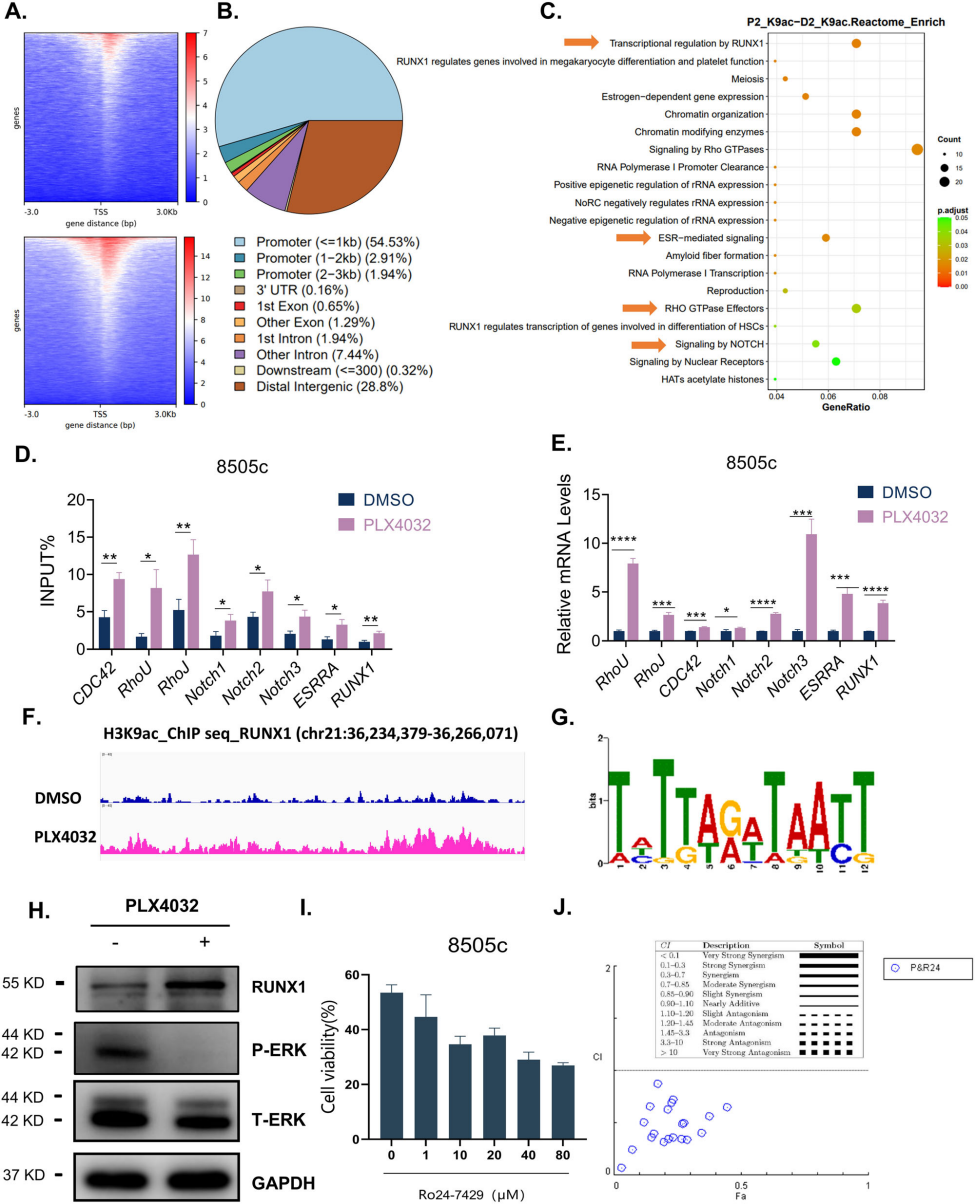
Enhanced H3K9ac facilitates pro-survival gene expression upon BRAFi in thyroid cancer cells. **A** ChIP-seq with H3K9ac antibody was performed after PLX4032 (5 μM, 24 h) treatment and the bining density of H3K9ac was visualized by deepTools; the heatmap presents the gene distance on the different H3K9ac bind peak before and after BRAF inhibition, ordered by signal strength. **B** Genome-wide distribution of up-regulated H3K9ac-bind peaks after PLX4032 (5 μM, 24 h) treatment compared to DMSO group. **C** Reactome enrichment analysis of the elevated H3K9ac binding peaks. **D** ChIP-qPCR assay of H3K9ac status of the indicated genes in 8505c cells after PLX4032 (5 μM, 24 h) treatment. **E** RT-qPCR detection of the indicated genes in 8505c cells after PLX4032 (5 μM, 24 h) treatment. **F** Genome browser tracks for RUNX1 at the representative target gene loci from the ChIP-seq analysis using Integrative Genomics Viewer (IGV) software. **G** Mapping on the RUNX1 motif logo determined by MEME motif analysis. **H** Western blot detection of RUNX1 in 8505c after PLX4032 (5 μM, 24 h) treatment. **I** 8505c was treated with PLX4032 (5 μM) in the absence or presence of different doses of RUNX1 inhibitor Ro24-7429 for 48 h, followed by viability measurement with CCK8. **J** The combination index (CI) was calculated by CompuSyn software, with CI <1 considering synergism. Data are presented as means ± s.d. Statistical significance was determined by Student’s *t* test in D and E, with *P* ≤ 0.05 considered significant. *P < 0.05, **P < 0.01, ***P < 0.005 and ****P < 0.001.

To uncover the role of BRAFi-induced H3K9 hyperacetylation, we performed H3K9ac ChIP-seq profiling in PLX4032-treated 8505c cells, revealing a marked increase in H3K9ac-enriched genomic regions compared to DMSO-treated controls (Fig. 6A). Further analysis demonstrated the preferential promoter localization (more than 50%) of differential H3K9ac peaks (Fig. 6B). To investigate the epigenetic modulatory impacts associated with H3K9ac changes, 393 differentially bound genes were selected for Reactome pathway analysis. This analysis identified significant enrichment in therapy resistance-associated pathways, including RhoGTPase signaling, Notch signaling, estrogen receptor signaling, and most prominently, RUNX1-mediated transcriptional regulation (Fig. 6C). To evaluate the functional relevance of these pathways, we selected a panel of candidate genes (RhoU, RhoJ, CDC42, Notch1-3, ESRRA, and RUNX1) linked to the identified pathways for validation. ChIP-qPCR confirmed significant H3K9ac enrichment at the promoter regions of these genes (Fig. 6D). Consistently, RT-qPCR revealed that PLX4032 treatment significantly upregulated transcription of these genes (Fig. 6E). Collectively, these findings together suggest that BRAFi upregulates H3K9ac, which epigenetically activates genes within multiple pro-survival pathways, thereby contributing to thyroid cancer cell survival.

Particularly, RUNX1 emerged as a focus of interest because it exhibited the most significant pathway-level alterations by ChIP-seq analysis. Intriguingly, although RUNX1 has been implicated in cancer progression and therapy resistance across multiple malignancies [22], its specific role in thyroid cancer and BRAFi remains poorly characterized. This prompted our investigation into RUNX1’s functional relevance. Genome browser track analysis revealed enhanced co-localization of RUNX1 and H3K9ac at chromatin loci (Fig. 6F), with the RUNX1 binding motif identified (Fig. 6G). Consistent with transcriptional activation, RUNX1 upregulation was confirmed at the protein level (Fig. 6H). Similarly, in BCPAP cells treated with PLX4032 or 8505c cells treated with PLX470, we also observed the increase of H3K9ac and RUNX1 (Fig. S4C, D), indicating that H3K9ac/RUNX1 activation is a general response to BRAFi in thyroid cancer. Upregulation of RUNX1 was also observed in PLX4032-resistant 8505c cells (Fig. S2E). Critically, co-treatment with the RUNX1 inhibitor Ro24-7429 demonstrated synergistic enhancement of PLX4032 cytotoxicity in 8505c cells (Fig. 6I, J). Collectively, these findings establish RUNX1 as a promising therapeutic target to improve BRAFi efficacy in thyroid cancer.

### RUNX1 is associated with aggressive clinicopathological features in thyroid cancer and functions as an oncogene

**Fig. 7:**
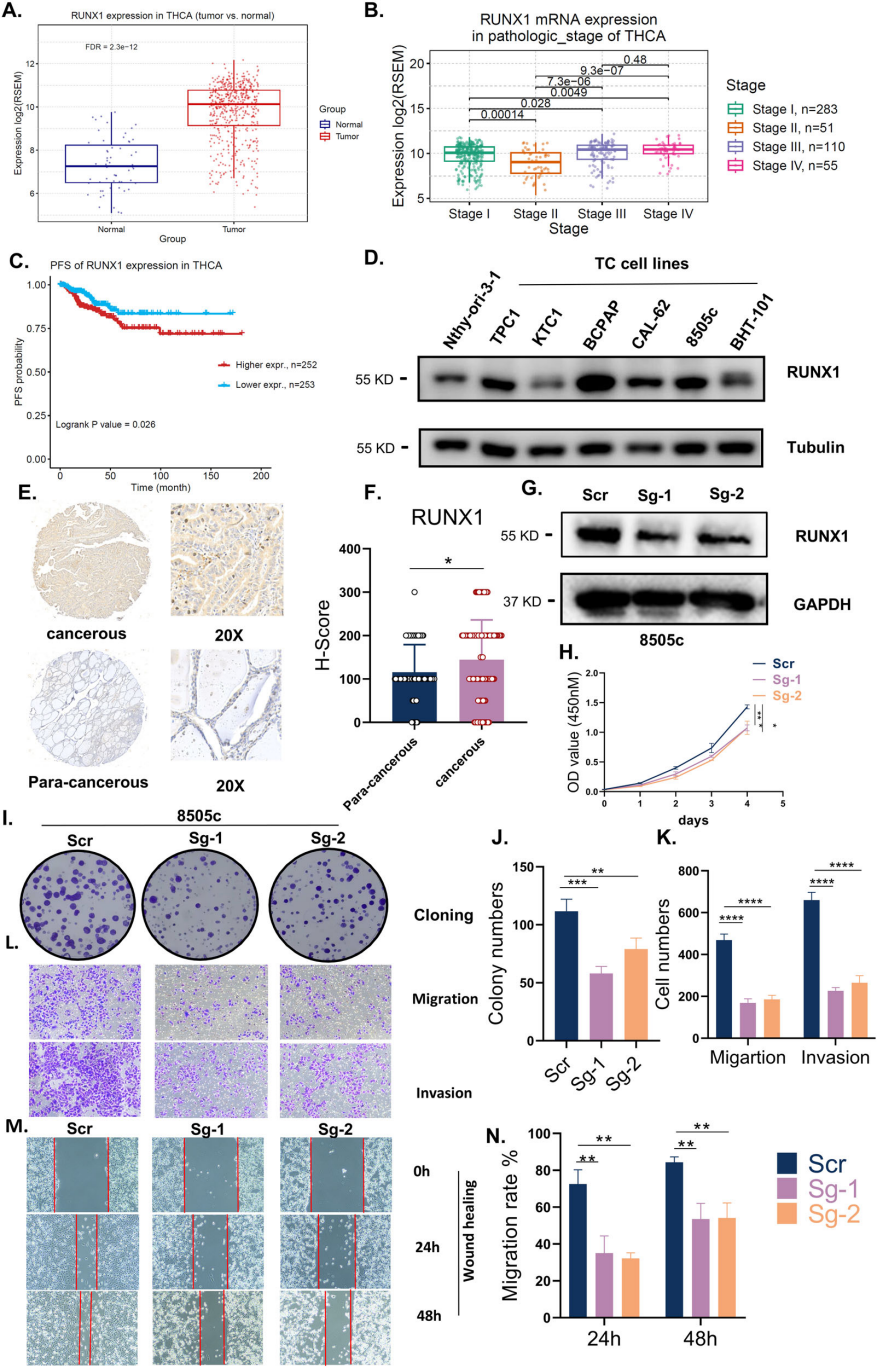
RUNX1 is associated with aggressive clinicopathological features in thyroid cancer and functions as an oncogene. **A** RUNX1 expression in paired samples of tumor tissues and nontumorous adjacent normal thyroid tissues from patients with thyroid cancer (THCA) in the TCGA database. **B** RUNX1 expression in samples of tumor tissues with different tumor stages from THCA in the TCGA database. **C** Kaplan–Meier analysis for PFS was performed according to RUNX1 expression levels. **D** Expression of RUNX1 in different thyroid cell lines, detected by western blot. **E** Representative immunohistochemical staining of RUNX1 in tumor and adjacent para-cancerous tissues of thyroid cancer patients (*n* = 88). **F** Statistical analysis of RUNX1 expression thyroid cancer and adjacent para-cancerous tissues. **G** Verification of RUNX1 knockdown efficiency by western blot in 8505c cells. **H** Cell growth curve of 8505c cells was detected by CCK-8 assay. Cell proliferation capacity was evaluated by colony formation assay (**I**) and corresponding statistical analysis (**J**). Cell migration and invasion capacity were evaluated by transwell assay (**L**) and corresponding statistical analysis (**K**). Cell migration capacity was evaluated by wound healing assay (**M**) after and corresponding statistical analysis (**N**). Data are presented as means ± s.d. Statistical significance was determined by Student’s *t* test in (**A**, **C**, **F**) ANOVA in (**B**, **H**, **J**, **K**, **N**) with *P* ≤ 0.05 considered significant. *P < 0.05, **P < 0.01, ***P < 0.005 and ****P < 0.001.

To systematically evaluate the role of RUNX1 in thyroid carcinogenesis, we first analyzed data from the Cancer Genome Atlas (TCGA) database. This analysis revealed significant upregulation of RUNX1 expression in thyroid cancer compared to normal tissues (Fig. 7A). Notably, RUNX1 expression was further elevated in patients with nodal metastasis (Fig. S5A). Moreover, RUNX1 levels were significantly higher in stage III/IV patients relative to stage I/II cases (Fig. 7B). Kaplan-Meier analysis demonstrated shorter progression-free survival (PFS; Fig. 7C) and reduced disease-free interval (DFI; Fig. S5B) in RUNX1-high patients. Pathway activity analysis indicated that high RUNX1 expression was associated with activation of the epithelial-mesenchymal transition (EMT) pathway and suppression of the DNA damage response (Fig. S5C, D). Furthermore, we assessed RUNX1 expression across a panel of thyroid cell lines. Consistent with the TCGA data, RUNX1 protein levels were markedly elevated in most thyroid cancer cells compared to the normal thyroid cell line Nthy-ori-3-1 (Fig. 7D). Additionally, immunohistochemistry (IHC) data from the Human Protein Atlas (HPA) database (4 thyroid carcinoma vs. 3 normal tissues) revealed moderate RUNX1 expression in 25% of carcinomas (1/4), whereas normal tissues uniformly exhibited low expression (3/3) (Fig. S5F). We also established a cohort of 88 papillary thyroid cancer patients, constructing a tissue microarray (TMA) from tumor and adjacent normal tissues. RUNX1 expression was quantified using H-scores, confirming significant upregulation in cancer tissues (Fig. 7E, F). Stratification of patients into high- and low-RUNX1 groups revealed that high RUNX1 expression correlated significantly with BRAF^V600E^ mutation and extrathyroidal invasion (Table 1). Overall, these results establish that RUNX1 is overexpressed in thyroid cancer and associated with aggressive clinicopathological features and poor prognosis.

**Table 1:** Association between RUNX1 expression and clinicopathological features in PTC.

	Number of patients	Low (*n* = 43)	High (*n* = 45)	*P* value
**Gender**				
Male	30	16 (37%)	14 (31%)	0.788
Female	58	27 (63%)	31 (69%)	
**Age (years)**				
≥55	17	10 (23%)	7 (16%)	0.36
<55	51	33 (77%)	38 (84%)	
**BRAFV600E mutation**				
Yes	71	31 (72%)	40 (89%)	**0.046**
No	17	12 (28%)	5 (11%)	
**Extrathyroidal invasion**				
Yes	36	12 (28%)	24 (53%)	**0.015**
No	32	31 (72%)	21 (47%)	
**Tumor size (cm)**				
≥1	77	37 (86%)	40 (89%)	0.687
<1	11	6 (14%)	5 (11%)	
**Multifocal**				
Yes	33	17 (40%)	16 (36%)	0.7
No	55	26 (60%)	29 (64%)	
**Bilaterality**				
Yes	27	13 (30%)	14 (31%)	0.929
No	61	30 (70%)	31 (69%)	
**Microscopic invasion**				
Yes	59	27 (63%)	32 (71%)	0.4
No	29	16 (33%)	13 (29%)	
**Lymph node metastasis**				
N0	25	14 (33%)	11 (24%)	0.399
N1	63	29 (67%)	34 (76%)	

Analyzed by *Chi-square* test.

Table 1 RUNX1 is associated with aggressive clinicopathological features in thyroid cancer patients.

To functionally validate the oncogenic activity of RUNX1, we first interrogated CRISPR-Cas9 screening data from the Cancer Dependency Map (DepMap). Analysis revealed that RUNX1 displayed a negative gene effect score (indicating impaired cell viability upon knockout) in the majority of thyroid cancer cell lines (10/14) (Fig. S5E), supporting its putative oncogenic function in thyroid tumorigenesis. To further corroborate these findings, we performed single guide RNA (sgRNA)-mediated knockdown of RUNX1 in 8505c cells. Following confirmation of the knockdown efficiency (Fig. 7G), we assessed phenotypes including proliferation, migration, and invasion. Consistent with DepMap, RUNX1 knockdown significantly attenuated proliferation and colony formation capacity (Fig. 7H–J), with concomitant reductions in migratory and invasive potential as evidenced by transwell and wound healing assays (Fig. 7K–N). Collectively, these functional studies establish RUNX1 as a critical driver of thyroid cancer progression.

## Discussion

Emerging evidence has demonstrated the association between dysregulated metabolism and drug response, enabling tumors to acquire adaptive plasticity and withstand therapeutic pressures such as chemotherapy [23–25]. While in thyroid cancer, the interplay between metabolism reprogramming and targeted therapy remains poorly characterized. We previously observed the reduction of glycolysis during BRAFi in ATC, suggesting that metabolic reprogramming may be linked to cellular adaptive responses to BRAFi in thyroid cancer [17]. In this study, we systematically demonstrate that BRAFi triggers a profound metabolic shift towards FAO, with pharmacological FAO inhibition via Thio significantly enhancing BRAFi sensitivity both in vitro and in vivo. Thio was originally developed as an antipsychotic agent [26]. In recent years, Thio has been evaluated in clinical trials for treating cancers such as acute myeloid leukemia [27], highlighting its significant clinical translational value. It can also function as an effective FAO inhibitor to overcome drug resistance, including prostate cancer and melanoma [28, 29]. Our research is consistent with these findings, strongly implying that Thio is a potential agent to sensitize BRAFi in thyroid cancer. Cheng et al. reported that the increased fatty acid uptake and FAO facilitate cell survival under cisplatin-induced oxidative stress in ovarian cancer [30]. In breast cancer, increased FAO has been demonstrated to mediate the development of tamoxifen resistance [31]. However, prior investigations have not fully resolved the mechanistic basis of FAO-mediated tumor survival. Our findings further advance the field by elucidating how FAO-driven acetyl-CoA surplus epigenetically reshapes the H3K9 acetylation landscape, thereby activating pro-survival transcriptional programs. To our knowledge, this represents the first mechanistic dissection of FAO-driven chromatin remodeling under drug pressure.

PGC1α and PPARα are two main regulators of FAO. In melanoma, Kovacs et al. reported increased PPARα-mediated FAO activation following MAPK inhibition [20]. PGC1α can serve as a PPARα obligate coactivator to achieve FAO activation [29]. Nevertheless, the regulatory role of PGC1α in BRAFi remains incompletely defined, especially in thyroid cancer. Interestingly, we observed remarkable upregulation of PGC1α upon BRAFi in our model; genetic knockdown coupled with pharmacological perturbation of PGC1α significantly attenuated FAO-related gene expression and metabolic flux, again underscoring its essential regulatory function in potentiating FAO in thyroid cancer. Intriguingly, thyroid cancer with BRAF^V600E^ mutation is negatively related to PGC1α expression and exhibits enhanced aerobic glycolysis rather than FAO phenotype [32, 33]. In our model, BRAFi disrupted this metabolic equilibrium through sequential activation of PGC1α and subsequent FAO upregulation. Together, these findings highlight the importance of BRAF^V600E^ dominating PGC1α expression and its connection with metabolism reprogramming. The activity of PGC1α can be regulated by both energy-sensing pathways (e.g., AMPK/SIRT) and inflammatory mediators such as NF-κB [34, 35]. During BRAFi, both AMPK and SIRT are activated [36, 37]. Concurrently, BRAF^V600E^ is also known to constitutively induce NF-KB activity [38], suggesting that BRAFi may activate PGC1α through these mechanisms. On the other hand, PGC1α is regulated at both transcriptional and translational levels. In melanoma, BRAF^V600E^ can transcriptionally regulate PGC1α expression via MITF (melanocyte lineage transcription factor) [39], however, MITF is specifically expressed in melanophores, leaves the mechanisms by which BRAF^V600E^ regulates PGC1α in thyroid cancer to be further elucidated. Thyroid-specific transcriptional factors such as HHEX are vital for thyroid differentiation and tumorigenesis [40]. Whether these factors can functionally mimic MITF in connecting BRAF^V600E^ with PGC1α requires future investigation. PGC1α mRNA itself is an important node of regulation as well; a recent report indicates that its translation can be regulated by the RNA-binding protein RBM43 [41]. While during BRAFi in melanoma, a dysregulated protein translation reprogramming is observed [42], whether BRAFi can modulate PGC1α expression at the post-transcriptional level is worth further exploring.

Acetyl-CoA, the principal product of FAO, serves dual roles as both a central metabolic intermediate and a key epigenetic modulator. By directly supplying acetyl groups for lysine acetylation, it thus couples cellular metabolic flux to epigenetic regulation [43]. Lysine acetylation is a widespread and versatile protein post-translational modification, including histone and non-histone targets. Yu et al. reported that FAO-derived acetyl-CoA increases acetylated-STAT3 in chemoresistant triple-negative breast cancer cells, thereby protecting cancer cells from apoptosis [44]. However, histone acetylation has a broader gene activation effect, and the link between FAO and histone acetylation has not been reported. Our findings reveal that FAO-generated acetyl-CoA surplus epigenetically reprograms the H3K9 acetylation landscape, which in turn activates a panel of pro-survival pathways. This establishes a novel metabolism-epigenetics axis explaining FAO-mediated adaptive drug response. On the other hand, histone acetylation dynamics are coordinated by counterbalanced activities of histone acetyltransferases (HATs) and deacetylases (HDACs) [45]. We previously observed the activation of EP300 (a HAT) during BRAFi [17], suggesting this HAT may orchestrate H3K9ac remodeling, though definitive mechanistic validation requires systematic interrogation in the future.

H3K9ac-specific ChIP-seq analysis identified transcriptional activation of several pro-oncogenic pathways after BRAFi, with the RUNX1 signaling pathway exhibiting the most profound enrichment. Further, RUNX1 inhibition synergizes with BRAFi to suppress thyroid cancer growth, highlighting its importance for promoting tumor survival. RUNX1 is recognized as a resistance mediator, including gemcitabine resistance in pancreatic cancer, imatinib response in chronic myeloid leukemia and other chemoresistance [22, 46]. Mechanistically, RUNX1 modulates ER stress via the BiP/PERK/eIF2α pathway to promote gemcitabine resistance and is linked to autophagy [22, 47]. In our previous study, BRAFi in thyroid cancer induced high levels of ER stress-mediated autophagy [9], which suggests that RUNX1 may confer BRAFi resistance via the ER stress-autophagy axis. RUNX1 transcriptionally regulates a series of cell fate-determining genes, including those involved in anti-apoptosis [48]. Our model revealed a perturbation of anti-apoptotic genes following BRAFi, further implicating that RUNX1 may promote cell survival via reprogramming the cellular transcriptional network. Furthermore, RUNX1 is an oncogenic driver in breast cancer and multiple malignancies [49, 50]. In thyroid cancer, Yang et al. performed genome profiling and RNA sequencing, indicating RUNX1 methylation as a cancer biomarker [51], however lacking systematic investigation and functional validation. Through functional experiments and integrative analysis, our study demonstrated that RUNX1 is highly expressed in thyroid cancer, relevant to poor prognosis, and functions as an oncogene. Our findings not only identify RUNX1 as a critical gene to promote tumor survival during BRAFi, but also provide an entry point for thyroid cancer development. Future studies will investigate RUNX1 function across expanded cohorts of clinical specimens and additional thyroid cancer cell lines to elucidate its role in thyroid carcinogenesis.

Drug resistance remains a major limiting factor for BRAFi efficacy, with resistance development representing an evolving and dynamic process. Through evaluations under both short-term and prolonged BRAFi exposure, we demonstrated that increased FAO dually acts as an acute adaptation and a sustained adaptive survival mechanism in BRAFi-resistant thyroid cancer. On the other hand, given the increasing clinical adoption of BRAFi+MEKi combination therapy, we expanded our findings to this dual-target model. By applying PDO models, we highlighted that targeting FAO can be an effective strategy to sensitize MAPK-targeted therapy.

Our work certainly has several limitations. First, as mentioned above, Thio is a multi-target agent. Beyond inhibiting FAO, it also antagonizes dopamine D2 receptors and potently inhibits the PI3K-Akt-mTOR pathway, exerting anti-angiogenic effects [52], indicating that off-target effects may confound the interpretation of relevant pharmacological assays. Second, although we clarified that both PGC1α and RUNX1 were activated following BRAFi, the precise mechanism by which BRAFi triggers PGC1α activation, as well as the downstream effects of RUNX1 upregulation, requires further exploration. Finally, the development of resistance is a gradual process that selects for tumor cells with adaptive survival advantages. Our work mainly focuses on the initial phase of resistance development. Although similar FAO enhancement was observed in long-term BRAFi-resistant 8505c cells, a more systematic investigation, including comprehensive metabolic profiling and mechanistic studies, is warranted in resistant cells. Furthermore, future studies will require comprehensive validation across a broader range of preclinical models, including more resistant cell lines, to robustly support our conclusions.

In conclusion, our study uncovered not only FAO-driven metabolic reprogramming during BRAFi in thyroid cancer but also elucidated the mechanistic basis of FAO-mediated tumor survival. FAO-derived acetyl-CoA accumulation reprograms H3K9 acetylation landscape, thereby activating pro-survival genes such as RUNX1. Pharmacological FAO inhibition potentiated BRAFi efficacy both in vitro and in vivo. Our findings illuminate a previously unrecognized paradigm of metabolism-epigenome crosstalk underlying adaptive resistance to BRAFi.

## Supplementary information

Clean supporting information

Supplemental Figure 1

Supplemental Figure 2

Supplemental Figure 3

Supplemental Figure 4

Supplemental Figure 5

origined WB

checklist

## Data Availability

Data are available from the corresponding author upon reasonable request.
