# Supplementary material for: Fatty acid oxidation drives acetyl-CoA-dependent H3K9ac reprogramming to promote adaptive resistance to BRAFV600E inhibition in thyroid cancer
Source: Cell Death Dis. 2026 Mar 20;17(1):329. doi: 10.1038/s41419-026-08575-7 (PMC13039272; doi:10.1038/s41419-026-08575-7)
Supplement: Supplementary file 1 — Clean supporting information [file 41419_2026_8575_MOESM1_ESM.docx]

**Supplementary material**

**Title:**

Fatty acid oxidation drives Acetyl-CoA-dependent H3K9ac reprogramming to promote adaptive resistance to BRAF^V600E^ inhibition in thyroid cancer

Xumeng Wang#, Jing Zhang#, Jimeng Yuan, Liping Wen, Tianxing Ying, Zehang Xu, Zheng Zhou, Shitu Chen, Quan Zhou, Jinghao Sheng, Chi Luo*, Lisong Teng*, Weibin Wang*.

1. **Supplemental methods and reagents**
   1. **Reagents**

The following reagents were applied in this study: Vemurafenib (stock concentration: 50mM, purchased from MedChemExpress, HY-12057), Dabrafenib (stock concentration: 10mM, purchased from MedChemExpress, HY-14660), PLX4720 (stock concentration: 10mM, purchased from MedChemExpress, HY-51424), Trametinib (stock concentration: 10mM, purchased from MedChemExpress, HY-10999), Thioridazine (stock concentration: 10 mM, purchased from AdooQ, A11119), SR-18292 (stock concentration: 10 mM, purchased from MedChemExpress, HY-101491), Etomoxir (stock concentration: 100 mM, purchased from AdooQ, A11415), Ro24-7429 (stock concentration: 10 mM, purchased from MedChemExpress, HY-19149), the above regents were dissolved in DMSO, Sodium acetate was purchased from angon Biotech, A601611. ^13^C labeled oleic acid was purchased from MedChemExpress (HY-N1446S2).

- 1. **Transfection and establishment of stable cells**

For stable PGC1α and RUNX1 knockdown cell establishment, the pLenti-SpBsmBI-sgRNA-Puro vector was used. Negative control sequences, PGC1α and RUNX1 sgRNA were generated by PCR and then cloned into the pLenti vector. HEK239T cells in well-growth conditions were chosen for transfection. A mixture of 2 μg targeted plasmid, 1.5 μg of Pspax plasmid, and 0.5 μg of pMD2.D plasmid in Opti-MEM reduced serum medium was transfected using Liposomal Transfection Reagent (YEASEN, Shanghai, China). Viral supernatant was collected at 48 h or 72 h post-transfection and filtered (0.45 nm pore size). The 8505c or WRO cells were infected in the presence of 8 μg/ml polybrene (Beyotime, Shanghai, China) for 48 h. then stable cells were selected by incubation with 1 μg/ml puromycin. The primers of corresponding sg-RNAs are listed in Supplementary material Table S1.

- 1. **Western blotting**

Cells were collected with radioimmunoprecipitation assay buffer containing protease inhibitor (biosharp, Anhui, China) and sonicated at 10% power (5’ on, 5’ off) for 6 cycles on ice. After centrifugation at 12,000 g for 10 min, the protein content in the supernatant was determined using the BCA protein assay kit (Beyotime, Shanghai, China). Protein samples were run on 8-15% sodium dodecyl sulfate-polyacrylamide gel electrophoresis and transferred to polyvinylidene fluoride (PVDF, Millipore) membrane. The membranes were incubated with primary antibodies at 4°C overnight, washed by TBST 3 times, and incubated with secondary antibodies at room temperature for 1 h. Finally, the signals were visualized with the chemiluminescence imaging system by Amersham Imager 600 (GE Healthcare Life Sciences, Connecticut, USA) or iBright 750 (Thermo Fisher Scientific, Massachusetts, USA). Primary antibodies used are listed in the Supplementary material Table S4.

- 1. **Total RNA extraction and quantitative real-time PCR**

Total RNA from thyroid cancer cells was isolated by TRIZOL reagent, and cDNA was reverse transcribed with Prime Script RT Master Mix (Takara, Kyoto, Japan). Quantitative real-time PCR was performed with SYBR Premix EX Taq II (Takara, Kyoto, Japan) in a StepOnePlus RT-PCR system (Roche, Basel, Switzerland). The amplified transcript level of each specific gene was normalized to β-actin or GAPDH by using 2^−ΔΔCt^ method. The sequences of primers used in this study are listed in the Supplementary material Table S2.

- 1. **Immunofluorescence**

Cells were first seeded into the 6-well culture and grew on glass coverslips, after different treatments, cells went triple PBS washing, followed by fixation in ice-cold 4% paraformaldehyde (PBS-based, 20 min at −20°C). Then incubated with pre-cooling 0.5% Triton-X-100 on ice for 5 min. After triple PBS rinses, samples were blocked with 5% goat serum (overnight, RT), followed by incubation with primary antibodies (4°C, overnight). Thereafter, the cells were incubated with the corresponding secondary antibodys (2 h, RT), and nuclei were counterstained with DAPI (15 min, RT). coverslips were mounted using anti-fade medium and imaged via confocal microscopy (Nikon, Tokyo, Japan) with standardized laser/exposure settings. Primary antibodies used are listed in the Supplementary material Table S4.

- 1. **Immunohistochemistry**

The immunohistochemical staining was performed according to a standard protocol, and a DAB substrate kit (ZSGB bio, Beijing, China) was used to visualize the signal. The staining scores were evaluated by two experienced pathologists as follows: the staining intensity was regarded as 0 (no staining); 1 (weak, light yellow); 2 (moderate, light brown); or 3 (strong, brown). The percentage of cells at each intensity level is determined across representative fields. The final H-score is derived using the formula: H-score=(% weak cells×1)+(% moderate cells×2)+(% strong cells×3). High expression levels of RUNX1 were defined as H-scores ≥ 200. Primary antibody of RUNX1 used is listed in the Supplementary material Table S4.

- 1. **Oxygen consumption rate analysis**

The cellular oxygen consumption rate was analyzed using the oxygen respirometry system (Strathkelvin, UK). Briefly, cells were first seeded into 6-well plates, after attachment, cells were replaced with substrate-limited medium (contain 5% dialyzed FBS and 100 μM BSA-conjugated oleic acid), then cells were treated with different drugs and following OCR analysis. After gentle digestion, cells were resuspended in 37°C preheated culture medium, adjusted to a density of 1-5 x 10^6^ cells/mL, and maintained in a 37°C water bath. After calibration of the oxygen respirometry system and reaching a stable baseline, 1 mL of cell suspension was transferred into the assay chamber. Record dissolved oxygen over time, when the oxygen consumption rate reached a plateau, the data was used for further analysis.

- 1. **Colony formation assay**

Cells were seeded in 6-well plates at a density of 1000 cells/well and cultured for 14 days to form colonies. Colonies were fixed with 4% paraformaldehyde for 15 min, then stained with 1% crystal violet for 15 min, and counted (diameters ≥ 1.5 mm).

- 1. **Cell proliferation assay**

Cells were seeded in 96-well plates at a density of 3000 cells/well. After attachment, cell proliferation was assessed daily using the Cell Counting Kit-8 (CCK-8, (YEASEN, Shanghai, China)) according to the manufacturer's instructions. Briefly, 10 μL of CCK-8 reagent was added directly to each well containing 100 μL of culture medium, followed by incubation at 37°C for 2 hours. The absorbance was then measured at 450 nm using a microplate reader.

- 1. **Transwell of migration and invasion assay**

Cell migration and invasion were evaluated using 24-well Transwell chambers with 8 μm pore membranes (Corning, New York, USA). Transfected cells (3 × 10^4^/well) were suspended in the serum-free medium and seeded into the upper chamber with Matrigel (for invasion assay) or without (for migration assay). The lower chamber contained the complete medium with 10% FBS. After 24 h of incubation, cells on the lower surface were fixed with 4% paraformaldehyde, stained with 1% crystal violet for 15 min, respectively, and imaged under a light microscope.

- 1. **Wound heal assay**

Briefly, transfected cells were seeded in 6-well plates and grown to 90% confluence, a sterile 200-μL pipette tip was used to create a linear scratch. After washing with PBS twice, cells were maintained in the serum-free medium for 24 h. The wounded areas were observed and captured at each time point.

**1.12. Cell apoptosis assessment**

Annexin V-Alexa Fluor 488/PI apoptosis detection kit (YEASEN, Shanghai, China) was used to assess the apoptosis of thyroid cancer cells. Briefly, the cells were plated in 6-well culture plates, after treated with drugs at indicated concentrations for 48 h, the cell were harvested, gently washed with cold PBS, and re-suspended in 500 μL of 1X binding buffer. Then, the cells were stained with 5 μL Annexin V-Alexa Fluor 488 and 10 μL PI in darkness for 10 min at room temperature. Stained cells were measured by flow cytometry (ACEA Biosciences, California, USA) and analyzed by the Novo Express software.

- 1. **Red oil O staining**

Briefly, cells were first seeded in 6-well plates, and the culture medium was replaced with fresh medium containing 500 μM oleic acid to induce lipid droplet formation on the second day. On the third day, cells were treated with either PLX4032 or DMSO for 48 h. Subsequently, the medium was aspirated, and cells were washed with PBS and following fixed using 4% paraformaldehyde for 15 min. After fixation, cells were incubated with the filtered red oil O solution for 30 min. Then cells were washed with PBS again. Images were captured under a microscope and absorbance was measured by microplate reader for lipid droplet quantification.

- 1. **Acetyl-CoA assay**

The cytosolic acetyl-CoA was measured by PicoProbe Acetyl CoA Assay Kit (ab87546) from Abcam (Cambridge, UK) according to the manufacturer’s instructions. Results were normalized to cell numbers.

- 1. **Flow cytometry of Mito-tracker**

Mito-tracker was detected according to the manufacturer’s instruction of Mito-Tracker Green kit (C1048) from Beyotime (Shanghai, China). Briefly, cells were seeded into 6-well plates, after 70-80% confluence was achieved, cells were treated with DMSO or vemurafenib for 24 h, thereafter cells were harvested, washed with PBS, then incubated with 1 ml of working solution of Mito-tracker for 30 min at 37°C. Pictures were captured by fluorescence microscope (Olympus Corporation, Tokyo, Japan), fluorescence was visualized using flow cytometry (ACEA Biosciences) and data was analyzed by the Novo Express software.

**2. Supplementary Figure Legends**

**Supplemental Figure. 1 metabolic reprogramming occurred during BRAFi in thyroid cancer cells**

1. volcano plot of the gene expression changes analyzed by RNA-seq.
2. FAO and glycolysis-related gene expression changes analyzed by RNA-seq.
3. RT-qPCR detection of OXPHOS genes in 8505c cells after PLX4032 (5 μM, 24 h) treatment.
4. Western blot detection of OXPHOS genes in 8505c cells after PLX4032 (5 μM, 24 h) treatment.
5. Mito-tracker Green staining in 8505c cells after PLX4032 (5 μM, 24 h) treatment.
6. immunofluorescence intensity of Mito-tracker Green staining was quantified by flow cytometer.

Data are presented as means ± s.d. Statistical significance was determined by Student’s t test in C and F, with P ≤ 0.05 considered significant. *P < 0.05, **P < 0.01, ***P < 0.005 and ****P < 0.001.

**Supplemental Figure. 2 FAO related genes were up-regulated both in PLX4032-resistant and BRAFi+MEKi treated thyroid cancer cells.**

**A.** IC_50_ (half maximal inhibitory concentration) of PLX4032 in resistant and parental 8505c cells was detected by CCK-8 kit.

1. RT-qPCR detection of FAO related genes in resistant and parental 8505c cells.
2. RT-qPCR detection of FAO related genes in in 8505c cell after 24h treatment of 5 μM BRAF^V600E^ inhibitor PLX4720.
3. RT-qPCR detection of FAO related genes in 8505c cells after 24h treatment of dabrafenib (D, 1 μM) plus trametinib (T, 250nM).
4. Western blot detection of RUNX1 in parental and resistant 8505c cells.

Data are presented as means ± s.d. Statistical significance was determined by Student’s t test in B-D, with *P* ≤ 0.05 considered significant. **P* < 0.05, ***P* < 0.01, ****P* < 0.005 and *****P* < 0.001.

**Supplemental Figure. 3 Activation of PGC1α drives FAO during BRAFi in thyroid cancer cells**

**A-B.** 8505c and WRO cells were treated with PLX4032 5 μM / PGC1α inhibitor SR-18292 10 μM independently or together for 24h, then fatty acid oxidation related genes were detected by RT-qPCR.

**C-D.** 8505c and WRO cells were treated with PLX4032 5 μM / PGC1α inhibitor SR-18292 10 μM independently or together for 24h, then acetyl-CoA content was measured by acetyl-CoA fluorometric assay kit (C) and OCR was measured by oxygen respirometry system (D).

Data are presented as means ± s.d. Statistical significance was determined by ANOVA analysis in A-D, with *P* ≤ 0.05 considered significant. **P* < 0.05, ***P* < 0.01, ****P* < 0.005 and *****P* < 0.001.

**Supplemental Figure. 4 FAO contributes to cell survival and H3K9ac/RUNX1 was activated during BRAFi in thyroid cancer cells**

1. BCPAP cells were treated with 5 μM BRAF^V600E^ inhibitor PLX4032 alone, 10 μM FAO inhibitor Thio alone or a combination of PLX4032 with Thio for 48 h, followed by cell number counting via cell counter and corresponding statistical analysis.
2. 8505c cells were treated with 5 μM PLX4720 alone, 10 μM Thio alone or a combination of PLX4720 with Thio for 48 h, followed by cell number counting via cell counter and corresponding statistical analysis.

**C-D.** BCPAP and 8505c cells were treated with 5 μM PLX4032 or 5 μM PLX4720 respectively, followed by western blot detection of H3K9ac and RUNX1.

Data are presented as means ± s.d. Statistical significance was determined by ANOVA analysis in A and B, with *P* ≤ 0.05 considered significant. ***P* < 0.01, ****P* < 0.005 and *****P* < 0.001.

**Supplemental Figure. 5 RUNX1 is highly expressed in thyroid cancer and associated with aggressive clinicopathological features**

1. RUNX1 expression in samples of tumor tissues with (N1) or without (N0) lymph node metastasis from THCA in the TCGA database.
2. Kaplan–Meier analysis for DFI was performed according to RUNX1 expression levels.

**C-D.** Potential effects of RUNX1 mRNA on pathway activity using the data from THCA in the TCGA database.

1. Gene effect scores of RUNX1 on thyroid cancer cells derived from DepMap database, Negative scores imply cell growth inhibition and/or death following gene knockout.
2. Representative IHC staining images of RUNX1 in the HPA database.

Data are presented as means ± s.d. Statistical significance was determined by Student’s t test in B-D, ANOVA analysis in A, with *P* ≤ 0.05 considered significant.

**Table S1 The primers of sg-RNAs**

| Gene name | primers (5'- 3') |
| --- | --- |
| *Sg-RUNX1#1-F* | CACCGCGTAGATGCCAGCACGAGCC |
| *Sg-RUNX1#1-R* | AAACGGCTCGTGCTGGCATCTACGC |
| *Sg-RUNX1#2-F* | CACCGATCGCTTTCAAGGTACTGGC |
| *Sg-RUNX1#2-R* | AAACGCCAGTACCTTGAAAGCGATC |
| *Sg-PGC1α-F* | CACCGGGCAATCCGTCTTCATCCAC |
| *Sg-PGC1α-R* | AAACGTGGATGAAGACGGATTGCCC |

**Table S2 Primers for RT-qPCR**

| Gene name | primers (5'- 3') |
| --- | --- |
| *CPT1A_F* | ATCAATCGGACTCTGGAAACGG |
| *CPT1A_R* | TCAGGGAGTAGCGCATGGT |
| *CPT1B_F* | ATCATGGCGTGGATGATGT |
| *CPT1B_R* | CCTCTCATGGTGAACAGCAA |
| *CPT2_F* | CATACAAGCTACATTTCGGGACC |
| *CPT2_R* | AGCCCGGAGTGTCTTCAGAA |
| *ACAA2_F* | CTGCTCCGAGGTGTGTTTGTA |
| *ACAA2_R* | GGCAGCAAATTCAGACAAGTCA |
| *ACADL_F* | AGGGGATCTGTACTCCGCAG |
| *ACADL_R* | CTCTGTCATTGCTATTGCACCA |
| *CAT_F* | TGGGATCTCGTTGGAAATAACAC |
| *CAT_R* | TCAGGACGTAGGCTCCAGAAG |
| *CRAT_F* | CCCTGGACCACTACCTGAAG |
| Gene name | primers (5'- 3') |
| *CRAT_R* | GGTCTTGAGCCACCACTCAG |
| *EHHADH_F* | AAACTCAGACCCGGTTGAAGA |
| *EHHADH_R* | TTGCAGAGTCTACGGGATTCT |
| *ACOX1_F* | TAACTTCCTCACTCGAAGCCA |
| *ACOX1_R* | AGTTCCATGACCCATCTCTGTC |
| *ABCD1_F* | TGACAGGACAGGAGAGCCAA |
| *ABCD1_R* | AGACTGTCGCTTTAGGCCCC |
| *PGC1α-F* | CAGCCTCTTTGCCCAGATCTT |
| *PGC1α-R* | TCACTGCACCACTTGAGTCCAC |
| *RUNX1_F* | ACCTCGAAACACAAGGCAGA |
| *RUNX1_R* | GCACTTGAGAGTCGACTGGA |
| *CDC42_F* | ACATCTGTTTGTGGATAACTCA |
| *CDC42_R* | GGGAGCCATATACTCTTGGA |
| *RhoU_F* | GACTCCAACTCTGTGACACTGC |
| *RhoU_R* | ATGAGGGGCTCACGACACT |
| *RhoJ_F* | ACAATGTCCAGGAGGAATGGG |
| *RhoJ_R* | TGTGCTCCGATCGCTTTTG |
| *Notch1_F* | CGCAGATGCCAACATCCAGG |
| *Notch1_R* | CCCAGGTCATCTACGGCGTTG |
| *Notch2_F* | ATGACTGCCCTAACCACAGG |
| *Notch2_R* | CTGGAGTACAGGAGGCGAAG |
| *Notch3_F* | CGTGGCTTCTTTCTACTGTGC |
| *Notch3_R* | CGTTCACCGGATTTGTGTCAC |
| *ESRRA_F* | GGCTGGAGCGAGAGGAGTATG |
| *ESRRA_R* | GGAGGAGCGGTAGCGTGAG |
| *NDUFA4_F* | ACTGTATGTGATGCGCTTGG |
| *NDUFA4_R* | CAGTTTGTTCCATGGCTCTG |
| *SDHA_F* | TGGGAACAAGAGGGCATCTG |
| *SDHA_R* | CCACCACTGCATCAAATTCATG |
| *CYC1_F* | CTACGGACACCTCAGGCAGT |
| *CYC1_R* | CAGGTCACTGGCACTCACAG |
| *COX5A_F* | GGGAATTGCGTAAAGGGATAA |
| *COX5A_R* | TCCTGCTTTGTCCTTAACAACC |
| *ATP5G1_F* | ATCATTGGCTATGCCAGGAA |
| *ATP5G1_R* | ATGGCGAAGAGGATGAGGA |
| *GAPDH_F* | CCTCAACTACATGGTTTACA |
| *GAPDH_R* | CTCCTGGAAGATGGTGAT |
| *β-ACTIN-F* | AGCGAGCATCCCCCAAAGTT |
| *β-ACTIN-R* | GGGCACGAAGGCTCATCATT |

**Table S3 Primers for ChIP-qPCR**

| Gene name | primers (5'- 3') |
| --- | --- |
| *ChIP-RUNX1_F* | GACGCTTGCTACAGACGTGA |
| *ChIP-RUNX1_R* | CCACTGCAGGGGTAGTGATT |
| *ChIP_CDC42_F* | ATCTCCTGACCTCGTGATCCG |
| *ChIP_CDC42_R* | TCACTGCAATCTCGACGTCC |
| *ChIP_RHOU_F* | GTCACCCTCTTGGGAGCTG |
| *ChIP_RHOU_R* | GAGGACCTGGAACACACGTT |
| *ChIP_RHOJ_F* | GCAGCGGATGAAACAGGAAG |
| *ChIP_RHOJ_R* | GTACTTCTGGGGAACGATGC |
| *ChIP_Notch1_F* | CTATGGCAGGCATTTTGGACT |
| *ChIP_Notch1_R* | GCTGATTTATTTCTCCACCACGA |
| *ChIP_Notch2_F* | GGGGCCAGGCTGTAGATTTT |
| *ChIP_Notch2_R* | CCATCTGACCAAGGGCCAAT |
| *ChIP_Notch3_F* | ACGAGTCCCGAACTCTGTA |
| *ChIP_Notch3_R* | GGGACCCTTGAGCGATTAG |
| *ChIP_ESRRA_F* | GCAACTTCCCAAAGGTGTGC |
| *ChIP_ESRRA_R* | AGCCTGCAGGTCCACTCTTA |

**Table S4 Anti-bodies list**

| Anti-bodies | Source | Catalog number |  |
| --- | --- | --- | --- |
| Anti-Acetyl-Histone H3 (Lys9) Rabbit mAb | PTM BIO | PTM-112RM |  |
|  |  |  |  |
| Anti-Acetyl-Histone H3 (Lys27) Rabbit mAb | PTM BIO | PTM-116RM |  |
|  |  |  |  |
| Histone H3 | Santa Cruz | sc-517576 |  |
| Anti-Phospho-p44/42 MAPK (Erk1/2) | Cell Signaling Technology | 8544s |  |
| Anti-p44/42 | Cell Signaling Technology | 4695T |  |
| MAPK (Erk1/2) |  |  |  |
| RUNX1 (middle) Polyclonal antibody | Proteintech | 25315-1-AP |  |
| ACOX1 Monoclonal antibody | Proteintech | 68017-1-Ig |  |
| CPT1A Polyclonal antibody | Proteintech | 15184-1-AP |  |
| Human oxidative phosphorylation immunoblotting kit | Proteintech | PK30006 |  |
| GAPDH Monoclonal antibody | Proteintech | 60004-1-Ig |  |
| β-Actin Monoclonal antibody | Proteintech | 66009-1-Ig |  |
| β-Tubulin Rabbit mAb | ABclonal | A12289 |  |
