# Supplementary figures and images for: Fatty acid oxidation drives acetyl-CoA-dependent H3K9ac reprogramming to promote adaptive resistance to BRAFV600E inhibition in thyroid cancer
Source: Cell Death Dis. 2026 Mar 20;17(1):329. doi: 10.1038/s41419-026-08575-7 (PMC13039272; doi:10.1038/s41419-026-08575-7)

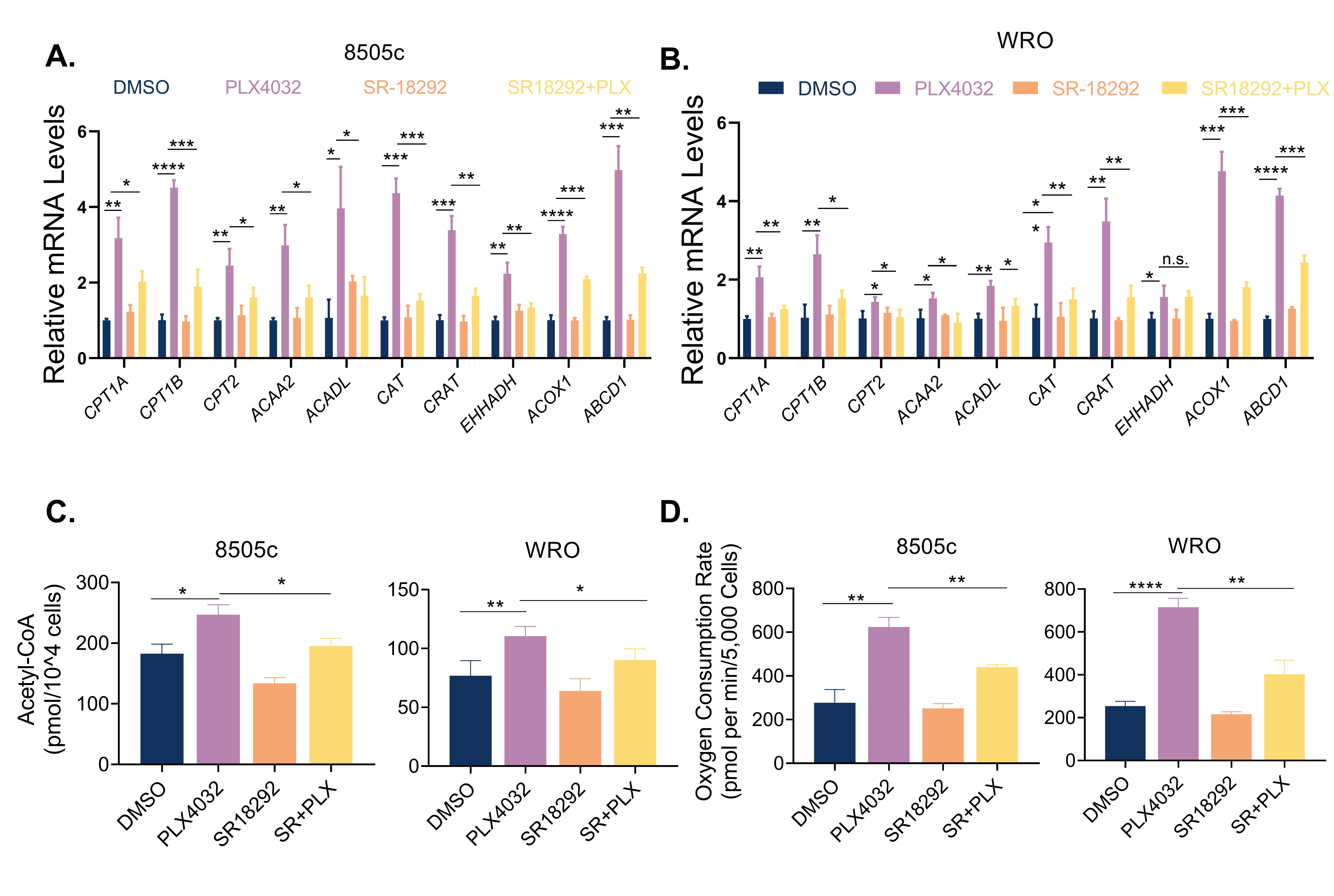

Supplement: Supplementary file 4 — Supplemental Figure 3 [file 41419_2026_8575_MOESM4_ESM.jpg]
