# Supplementary material for: Fatty acid oxidation drives acetyl-CoA-dependent H3K9ac reprogramming to promote adaptive resistance to BRAFV600E inhibition in thyroid cancer
Source: Cell Death Dis. 2026 Mar 20;17(1):329. doi: 10.1038/s41419-026-08575-7 (PMC13039272; doi:10.1038/s41419-026-08575-7)

Fig. 1E

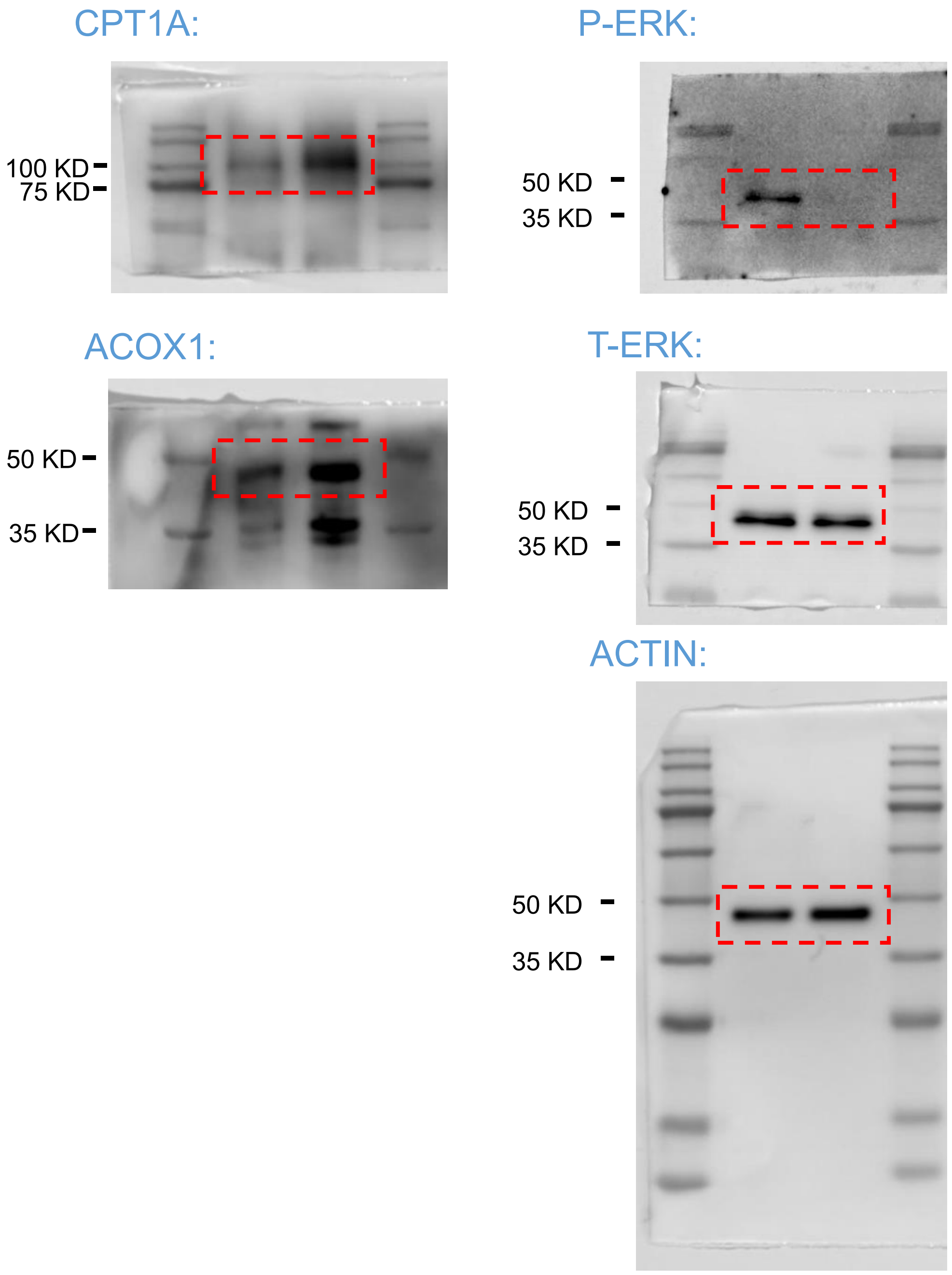

CPT1A:
100 KD
75 KD
P-ERK:
50 KD
35 KD
ACOX1:
50 KD
35 KD
T-ERK:
50 KD
35 KD
ACTIN:
50 KD
35 KD

Fig. 5A

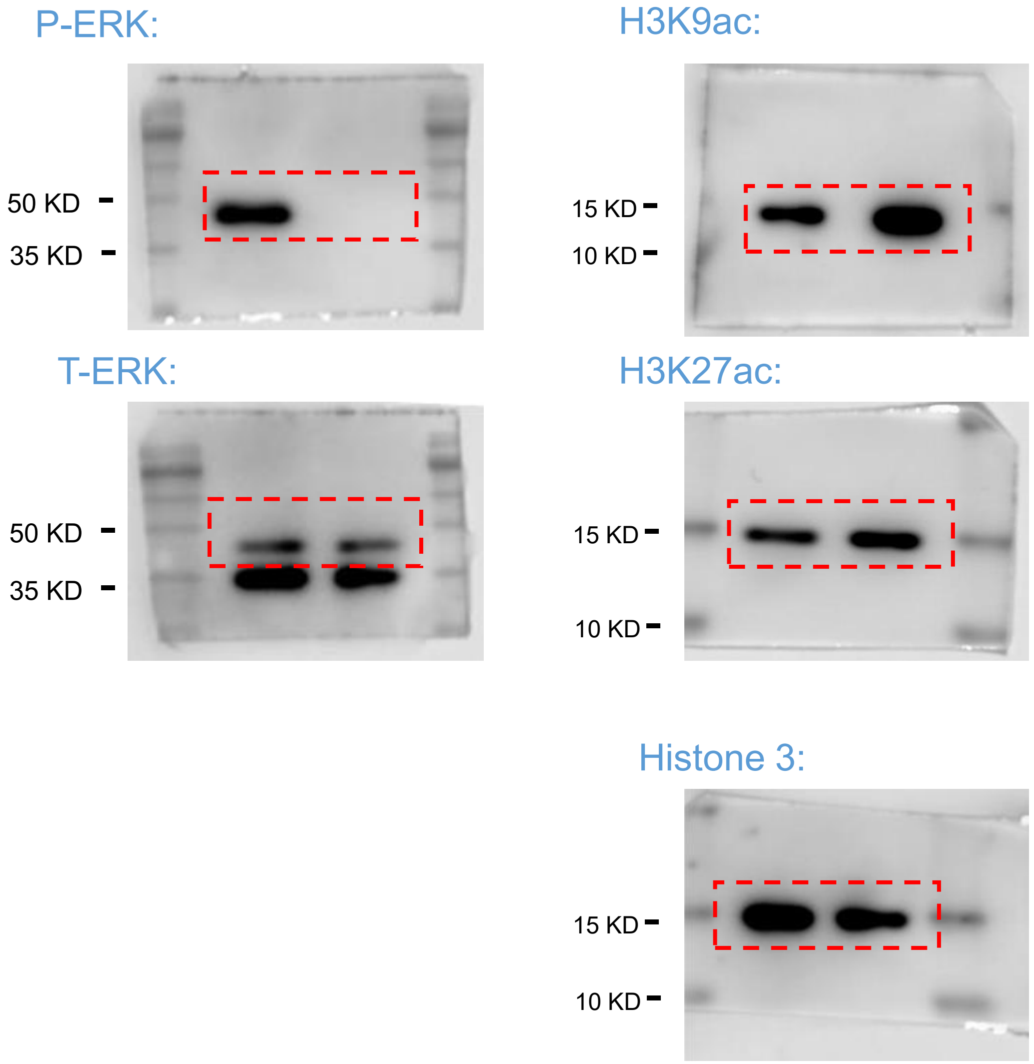

P-ERK:
50 KD
35 KD
H3K9ac:
15 KD
10 KD
T-ERK:
50 KD
35 KD
H3K27ac:
15 KD
10 KD
Histone 3:
15 KD
10 KD

Fig. 5E

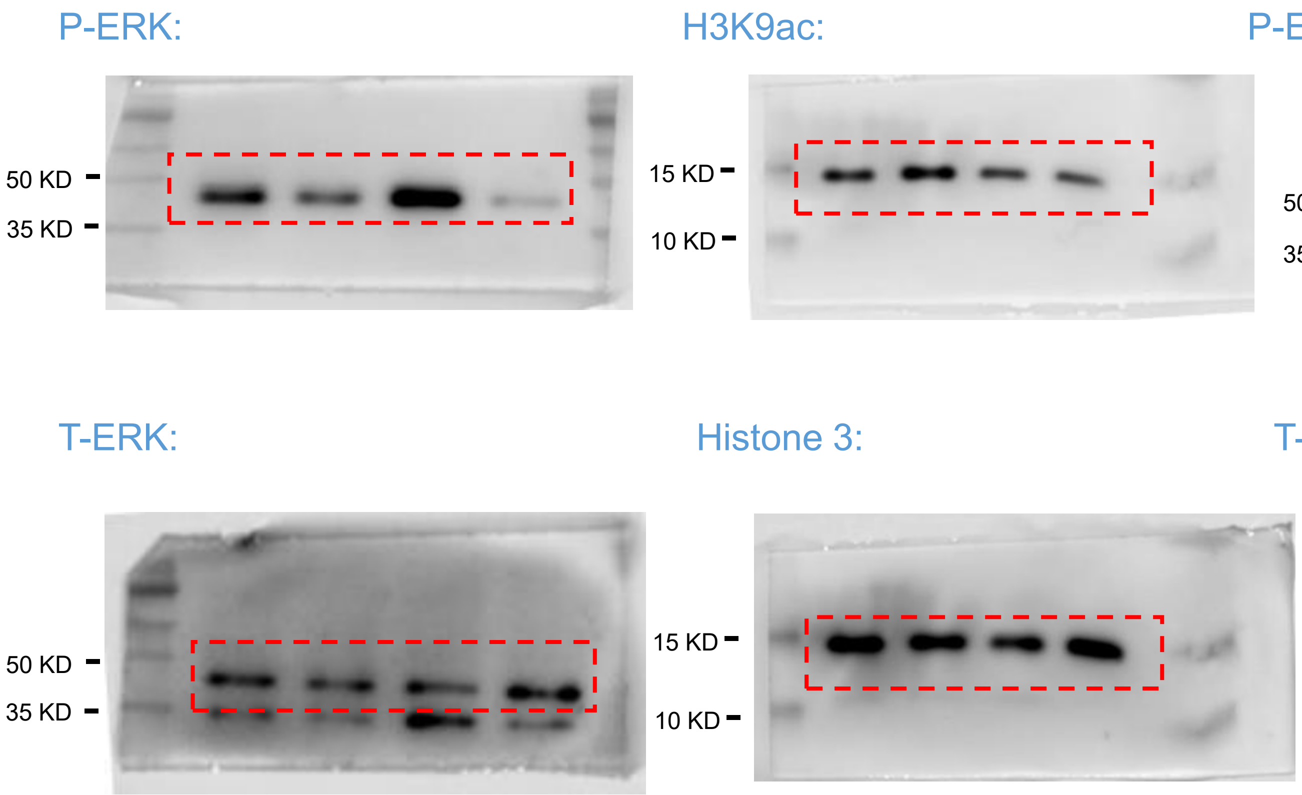

P-ERK:
50 KD
35 KD
H3K9ac:
15 KD
10 KD
T-ERK:
50 KD
35 KD
Histone 3:
15 KD
10 KD

Fig. 5H

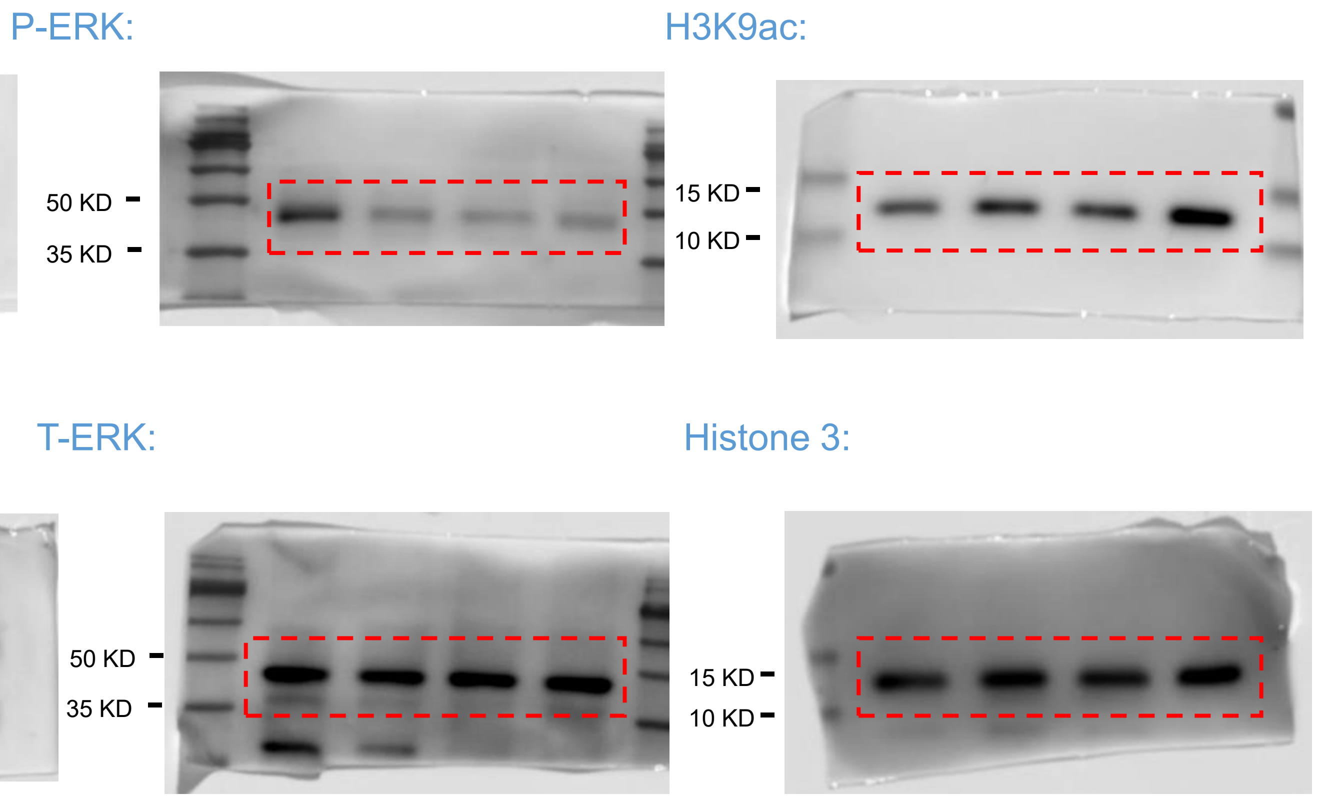

P-ERK:
50 KD
35 KD
H3K9ac:
15 KD
10 KD
T-ERK:
50 KD
35 KD
Histone 3:
15 KD
10 KD

Fig. 6H

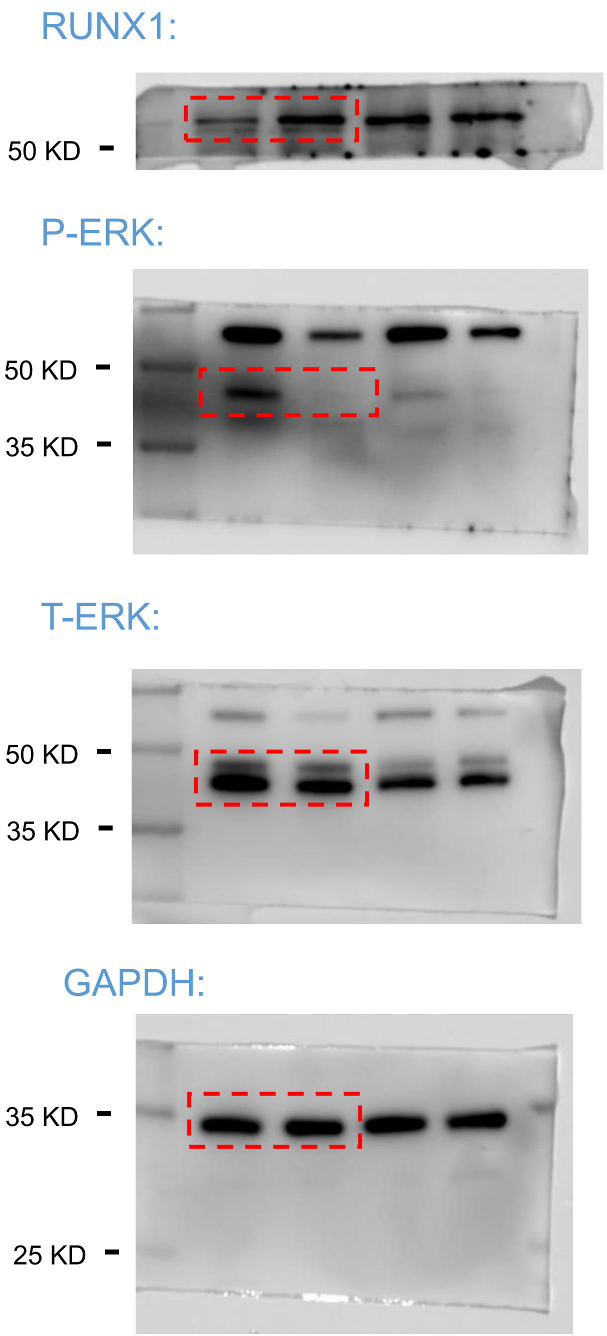

Fig. 7D

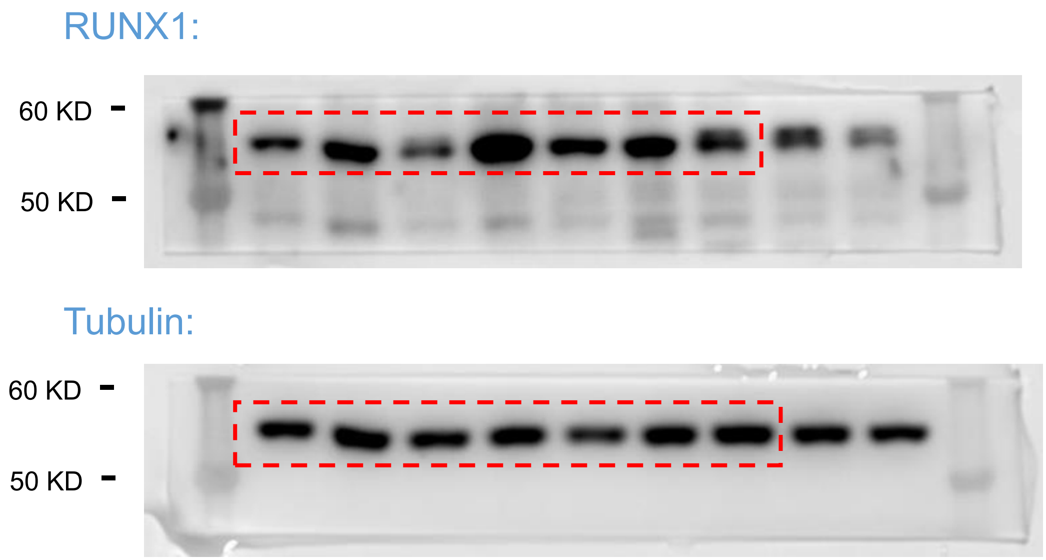

Fig. 7H

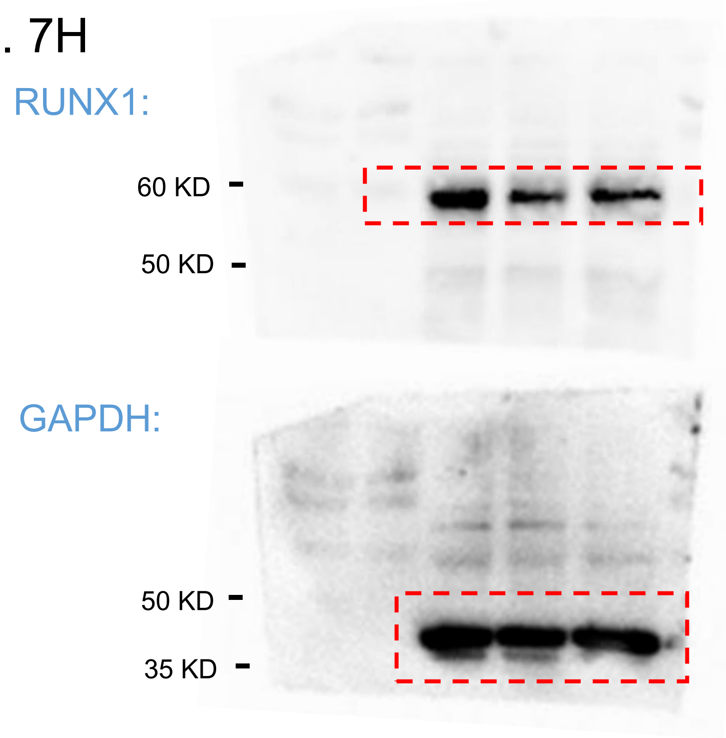

Fig. S1D

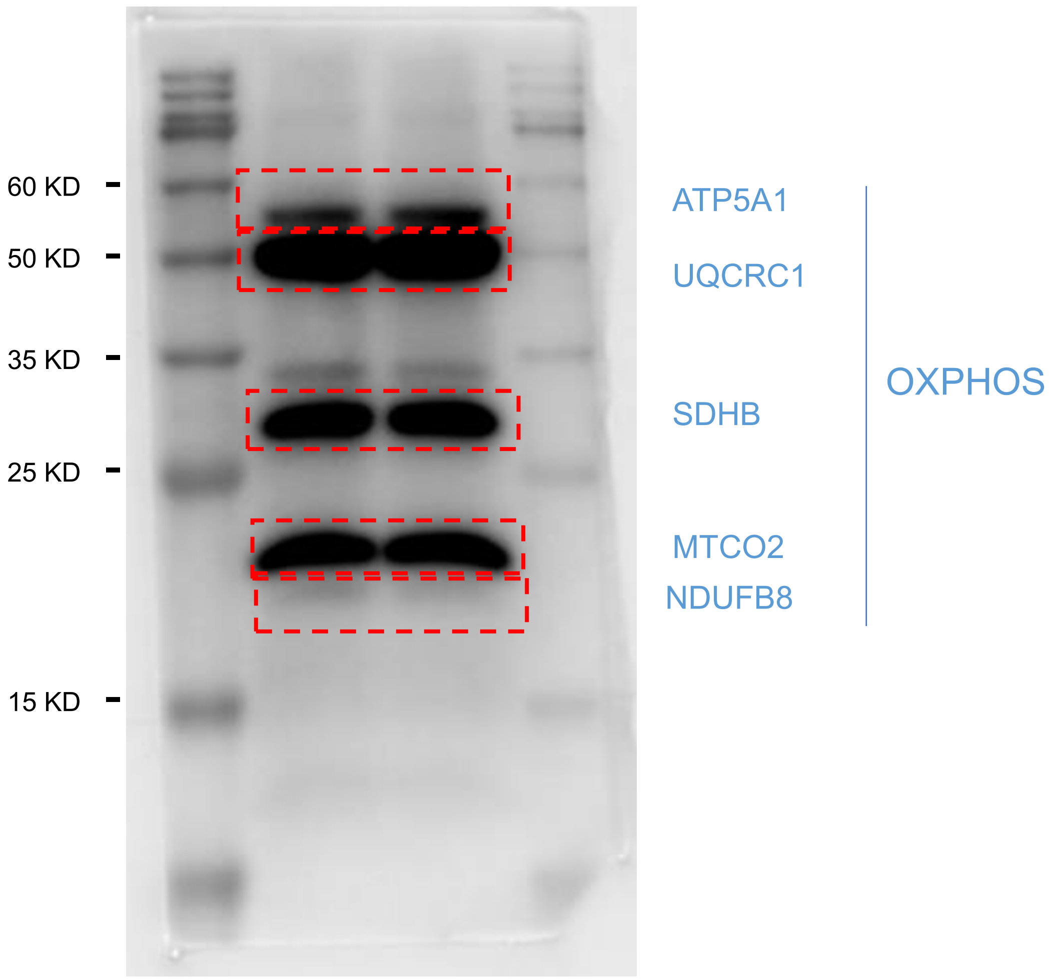

Fig. S2E

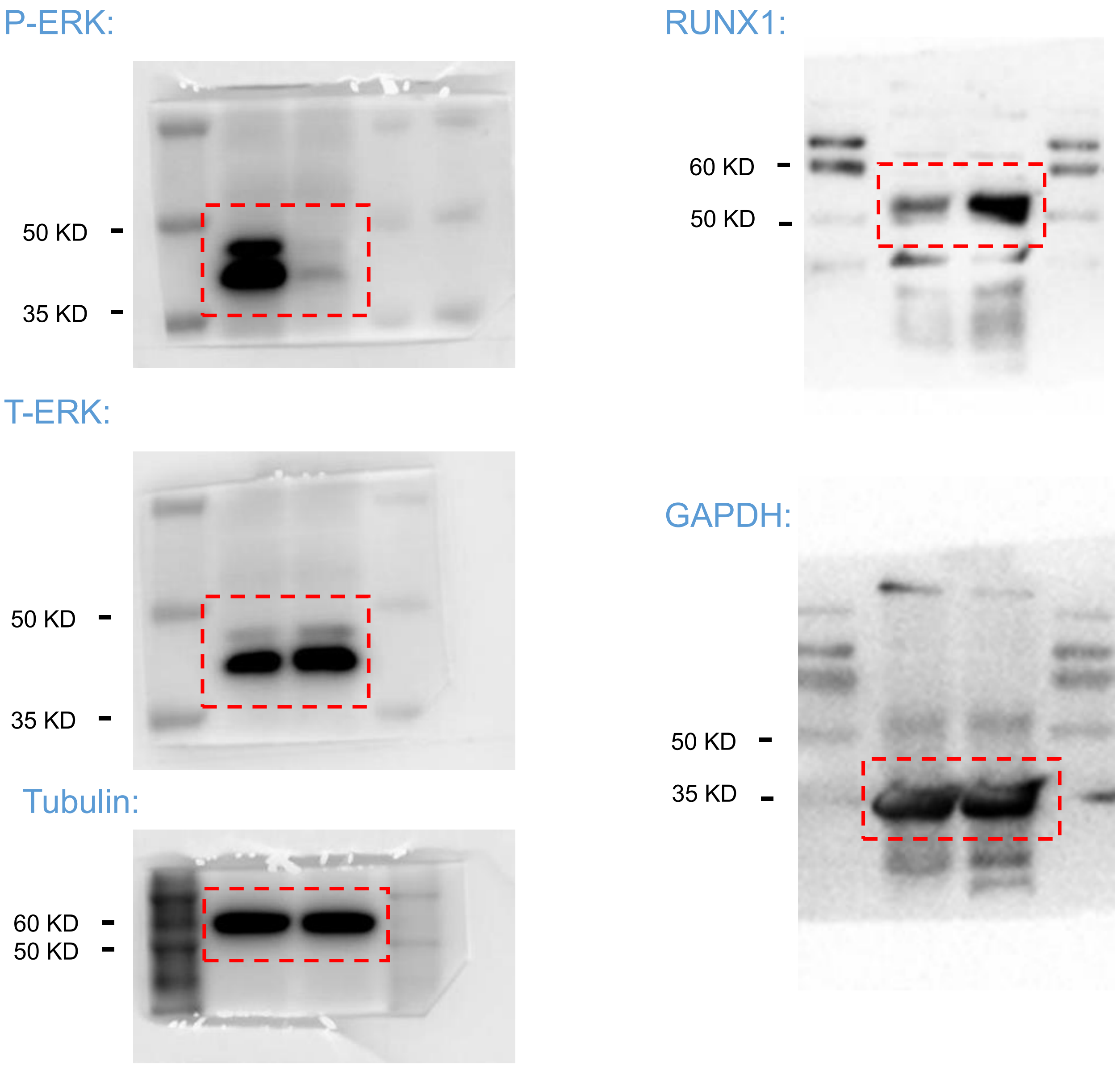

Fig. S4C

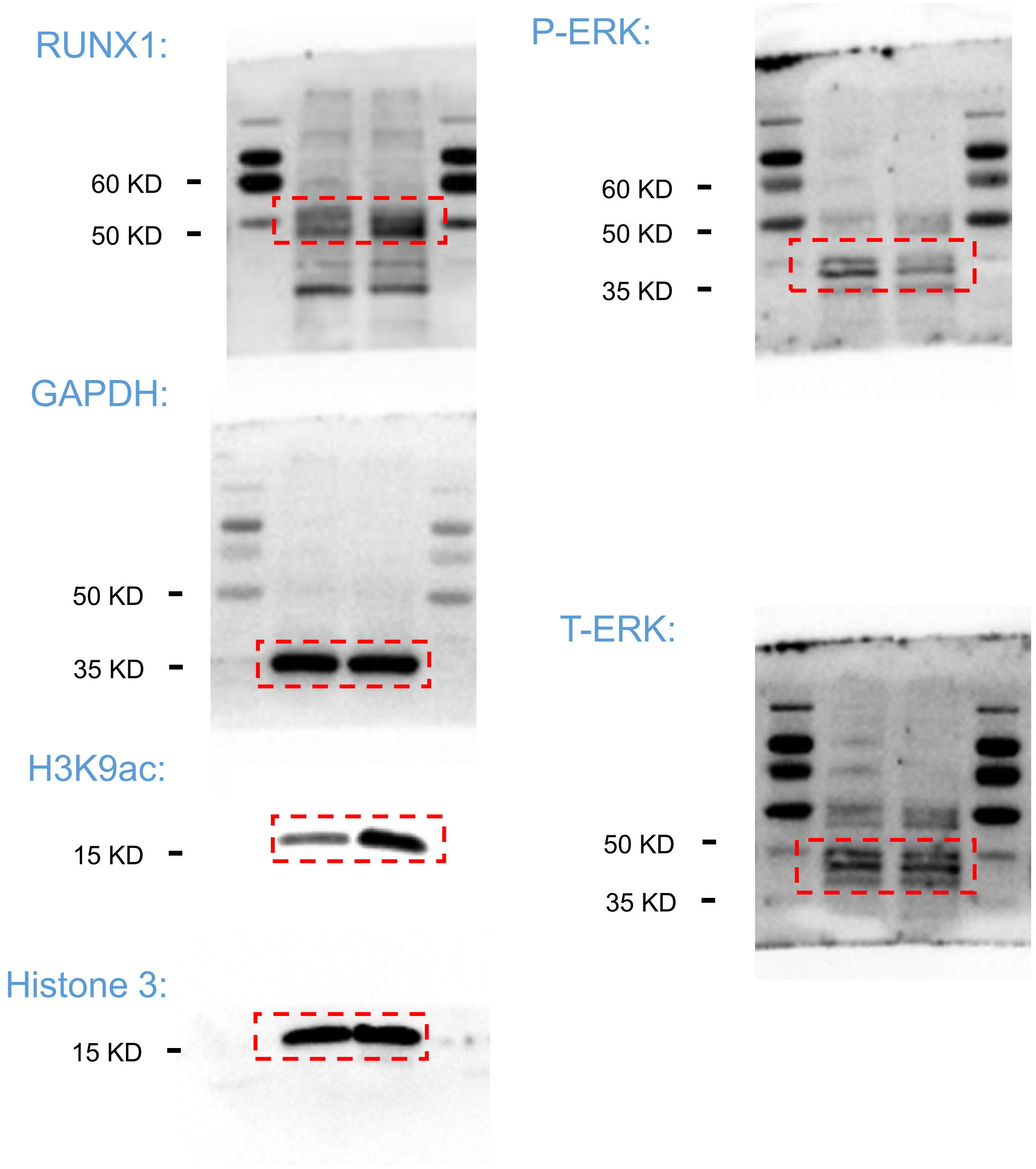

Fig. S4D

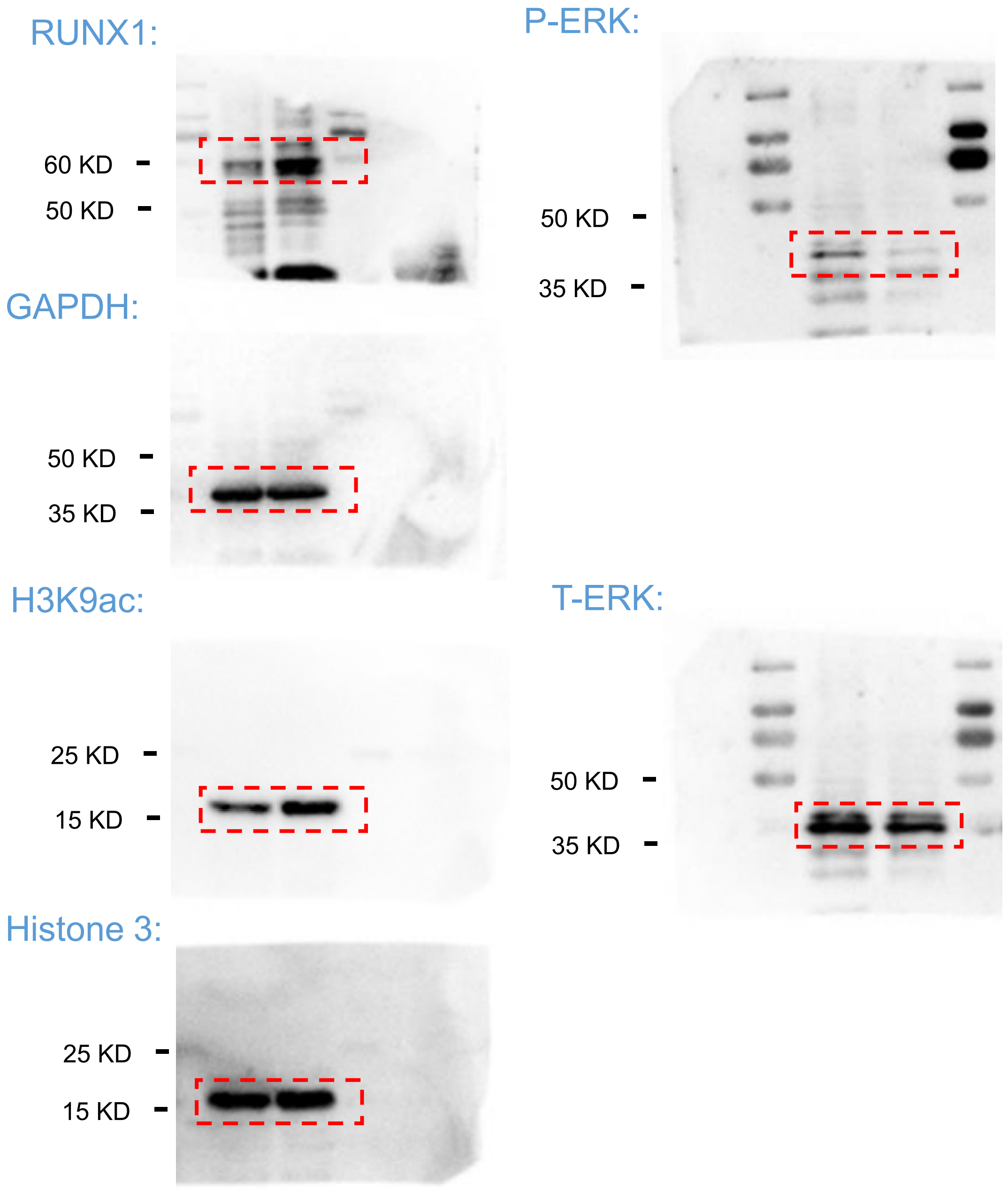

Supplement: Supplementary file 7 — origined WB [file 41419_2026_8575_MOESM7_ESM.pdf]
